# Phycobilisome’s Exciton Transfer Efficiency Relies on an Energetic Funnel Driven by Chromophore–Linker Protein Interactions

**DOI:** 10.1021/jacs.3c01799

**Published:** 2023-05-18

**Authors:** Siddhartha Sohoni, Lawson T. Lloyd, Andrew Hitchcock, Craig MacGregor-Chatwin, Ainsley Iwanicki, Indranil Ghosh, Qijie Shen, C. Neil Hunter, Gregory S. Engel

**Affiliations:** † Department of Chemistry, James Franck Institute and Institute for Biophysical Dynamics, Pritzker School of Molecular Engineering, The University of Chicago, Chicago, Illinois 60637, United States; ‡ School of Biosciences, University of Sheffield, Sheffield S10 2TN, U.K.

## Abstract

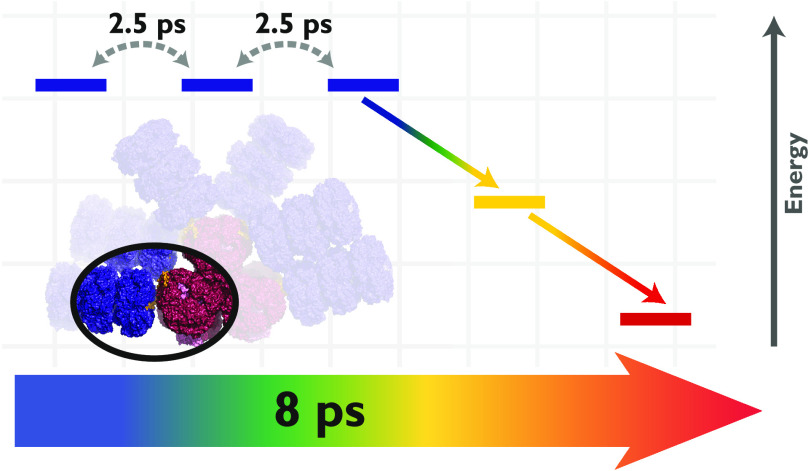

The phycobilisome is the primary light-harvesting antenna in cyanobacterial and red algal oxygenic photosynthesis. It maintains near-unity efficiency of energy transfer to reaction centers despite relying on slow exciton hopping along a relatively sparse network of highly fluorescent phycobilin chromophores. How the complex maintains this high efficiency remains unexplained. Using a two-dimensional electronic spectroscopy polarization scheme that enhances energy transfer features, we directly watch energy flow in the phycobilisome complex of *Synechocystis* sp. PCC 6803 from the outer phycocyanin rods to the allophycocyanin core. The observed downhill flow of energy, previously hidden within congested spectra, is faster than timescales predicted by Förster hopping along single rod chromophores. We attribute the fast, 8 ps energy transfer to interactions between rod-core linker proteins and terminal rod chromophores, which facilitate unidirectionally downhill energy flow to the core. This mechanism drives the high energy transfer efficiency in the phycobilisome and suggests that linker protein–chromophore interactions have likely evolved to shape its energetic landscape.

## Introduction

**Figure 1:**
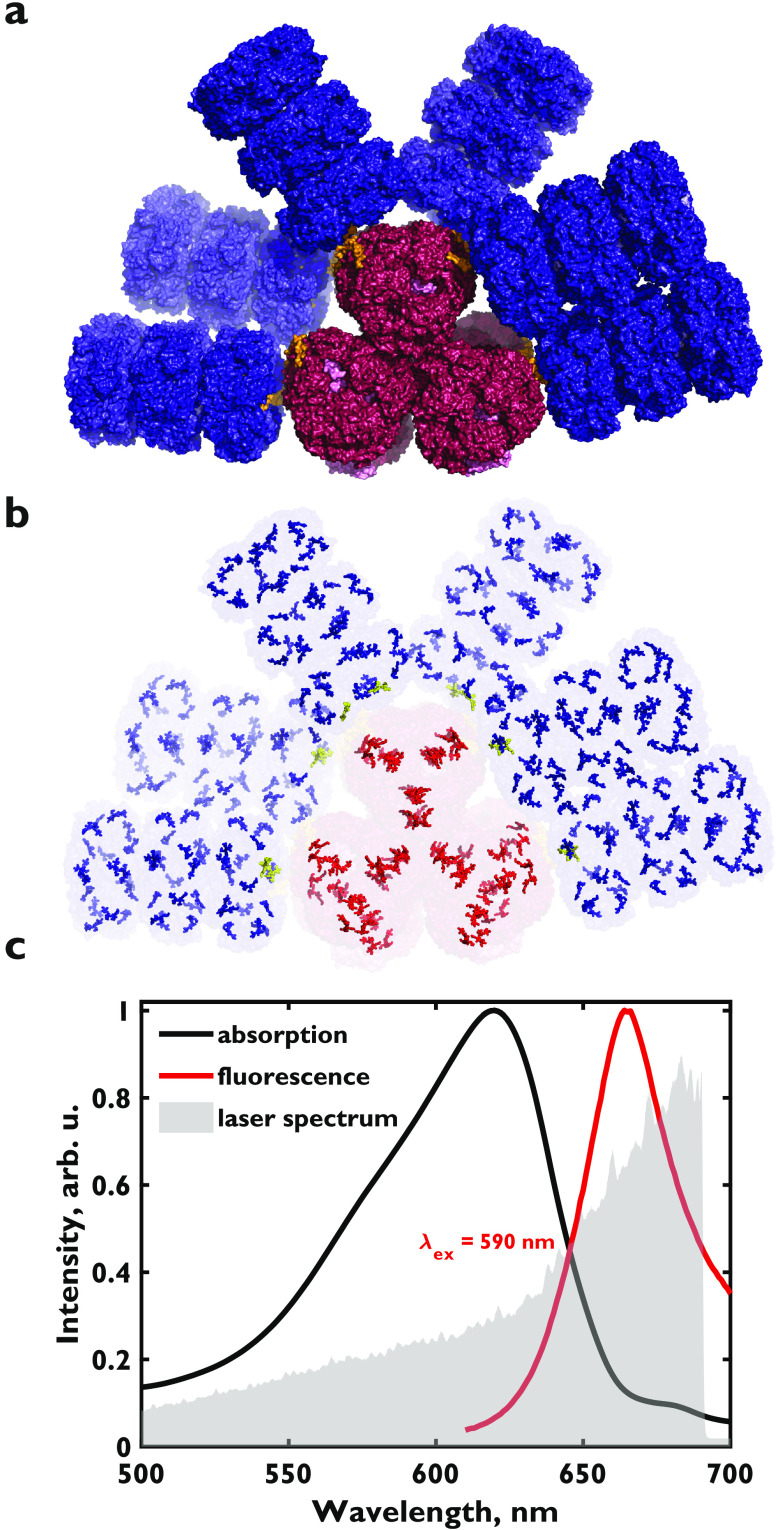
(a) Cryo-electron microscopy structure of the *Synechocystis* sp. PCC 6803 phycobilisome rendered using coordinates from Kerfeld and co-workers:^15^ six hexameric C-phycocyanin rods (blue) are assembled on the three lateral allophycocyanin cores (red). (b) Arrangement of phycocyanobilin chromophores in the phycobilisome (blue) with terminal rod chromophores (gold) strongly associated with the CpcG rod-core linker protein, and core allophycocyanin chromophores (red). (c) Absorption (black) and fluorescence (red) spectra overlaid with the laser spectrum (gray) used in our broadband two-dimensional spectroscopy experiments.

Cyanobacteria are oxygenic photosynthetic microorganisms. They produce about 40% of the world’s oxygen and were responsible for the Great Oxygenation Event of our planet.^1−3^ During oxygenic photosynthesis in cyanobacteria, plants, and algae, solar energy absorbed by large networks of antenna pigments migrates to reaction centers, where it is converted to a charge separation. For example, excitons created in the C_2_S_2_M_2_–LHCII complex of plants rapidly transfer to the reaction centers in photosystem II (PSII).^4,5^ In cyanobacteria, the ca. 5–8 MDa phycobilisome complex composed of multiple phycocyanin (PC) and allophycocyanin (APC) protein subunits serves as the primary light-harvesting antenna. It supplies excitations to both photosystems with near-unity efficiency.^6,7^ The phycobilisome complex from the model cyanobacterium *Synechocystis* sp. PCC 6803 consists of six PC hexamer rods attached by a linker protein onto a core made of three lateral APC hexamer assemblies (Figure 1a). The core sits atop the photosystems and funnels excitations to them.^6^ The colorless linker proteins do not participate in light harvesting but provide structural integrity to this megacomplex by connecting the rods to the core.^4,8−15^ In high light fluences, the orange carotenoid protein (OCP) attaches to the phycobilisome core to efficiently quench excitations before they reach the photosystems.^15^

Despite seemingly deleterious slow exciton hopping over the sparse arrangement of highly fluorescent chromophores, the complex maintains near-unity exciton transfer efficiency.^6,7^ As detailed in numerous studies, the mechanisms yielding this high transfer efficiency are not well known.^4,8−13^ Electron microscopy (EM) advances in recent years have elucidated the detailed structure of these megacomplexes and provided many clues about the underlying mechanisms.^8,12,14−17^ However, progress in resolving energy transfer dynamics using time-resolved spectroscopy has been slower owing to massive spectral congestion from hundreds of phycobilisome pigments.^18−26^ Small energetic separations in the donor and acceptor chromophores in the network confound isolation of signal kinetics from individual pigments or complexes, hiding the principles driving high transfer efficiencies that operate on the supercomplex or quaternary level. An alternative to using spectral resolution to reveal excitonic pathways is to use the different dipole directions of the linear phycocyanobilin chromophores in the complex through polarization-dependent spectroscopy. Conveniently, the bilins in the core proximal to the rods sit at about 60° to those in the rods.

To selectively obtain signals associated with energy transfer along the chromophore network, we perform polarization-controlled broadband two-dimensional electronic spectroscopy (2DES) on the cyanobacterial phycobilisome complex. Using a previously reported polarization sequence^27,28^ in 2DES, we suppress signals from interactions with parallel transition dipole moments removing the spectral congestion, dynamic Stokes shift of chromophores, and excited state absorption in this antenna. The suppression allows us to watch energy transfer selectively and directly from the rods of the phycobilisome to the inner core. We find that downhill energy transfer along the complex occurs on a much faster timescale (∼8 ps) than suggested by previous time-resolved experiments and Förster calculations. We attribute the fast energy transfer to key interactions between chromophores and the aromatic residues of the CpcG linker protein that connects PC rods to the lateral APC core. These interactions lower the energy of chromophores that form the rod-to-core connection in the excitonic pathway. This redshift has been characterized extensively with fluorescence measurements by Sauer and Pizarro.^29^ They observe an emission redshift of about 150 cm^–1^ from the terminal phycocyanobilin chromophores in PC rods.^29^ Multiple cryo-EM structures show that interactions between terminal chromophores and linker proteins are conserved across phycobilisomes of cyanobacterial and red algal species.^8,12,14−17^ This redshift of the terminal chromophore promotes unidirectional downhill energy flow and minimizes exciton random walk along isoenergetic chromophores, in turn minimizing the probability of fluorescence and trapping. These results unearth a previously hidden photosynthetic design principle operating on the quaternary or supercomplex level that supports robust near-unity exciton transfer efficiency in oxygenic photosynthesis. We also find that upon reaching the core, the excitations remain in the higher-energy core proteins for at least 800 ps, which is the majority of the phycobilisome fluorescence lifetime. Recent photoprotected cryo-EM structures show that these proteins are sites of photoprotection through OCP attachment.^15^ The structures suggest that our observed long stay of excitons in the higher-energy core proteins provides robust photoprotection opportunities to the antenna.

Phycobilisome absorption is tuned to 600–660 nm, which is outside of the main chlorophyll absorption bands (Figure 1c). Enhanced absorption in this region of the solar spectrum has in part driven cyanobacteria to become prolific and widespread photosynthesizers in marine and terrestrial habitats.^30−32^ Unlike most other light-harvesting antennas, the spatial arrangement of about 300–400 chromophores in this complex is relatively sparse (Figure 1b) with the average distance between neighboring chromophores exceeding 2.5 nm.^5,8,9^ Therefore, incoherent Förster-type excitation energy transfer (FRET) is the dominant energy transfer mechanism between pigments.^19,33−36^ The primary light-absorbing pigments in phycobilisomes are derivatives of open-chain tetrapyrrole molecules bound covalently to the protein backbone. These chromophores have a significantly higher fluorescence quantum yield in vitro than chlorophyll derivatives found in other antennas. In coherent time-resolved spectra, the phycobilisome shows a broad photoinduced absorption (PIA) feature suggested to arise from an electrochromic shift coupling^21,37,38^ of excited- and ground-state chromophores, which masks the red-most absorption and complete emission profile.^18,21,39^ Previous time-resolved studies have suggested that excitations created in the rods travel downhill to the higher-energy ApcA and ApcB (hereafter called APC_660_ for their emission maxima) core proteins on the 30–50 ps timescale and from APC_660_ to the core terminal emitters bound to ApcD, ApcE, and ApcF (hereafter called APC_680_) proteins, on the 100 ps timescale before fluorescing with ca. 1–2 ns lifetime in detached complexes.^15,19,21,22,26^ APC_680_ terminal emitters transfer excitations to the photosystems that lie beneath the phycobilisomes.^40,41^ A new study by Beck and co-workers uses global analysis of 2DES data and suggests that excitons travel to the core in 13 ps.^20^ The exact timescale of rod-to-core transfer remains a topic of debate and differs in different global analyses. Many of the FRET steps along the way to the allophycocyanin core have time constants longer than 10 ps.^33^ A bottleneck is predicted at the rod-to-core transfer step due to the large interchromophore distance, which makes backward hopping into the rod more favorable^15^ and confounds our understanding of the high transfer efficiency of the complex. Here, we investigate the basis for high energy transfer efficiency in the phycobilisome by directly monitoring energy flow in the phycobilisome complex of *Synechocystis* sp. PCC 6803 using polarization-controlled 2DES.

## Results

### Two-Dimensional Electronic Spectroscopy with Identically Polarized Pulses

**Figure 2:**
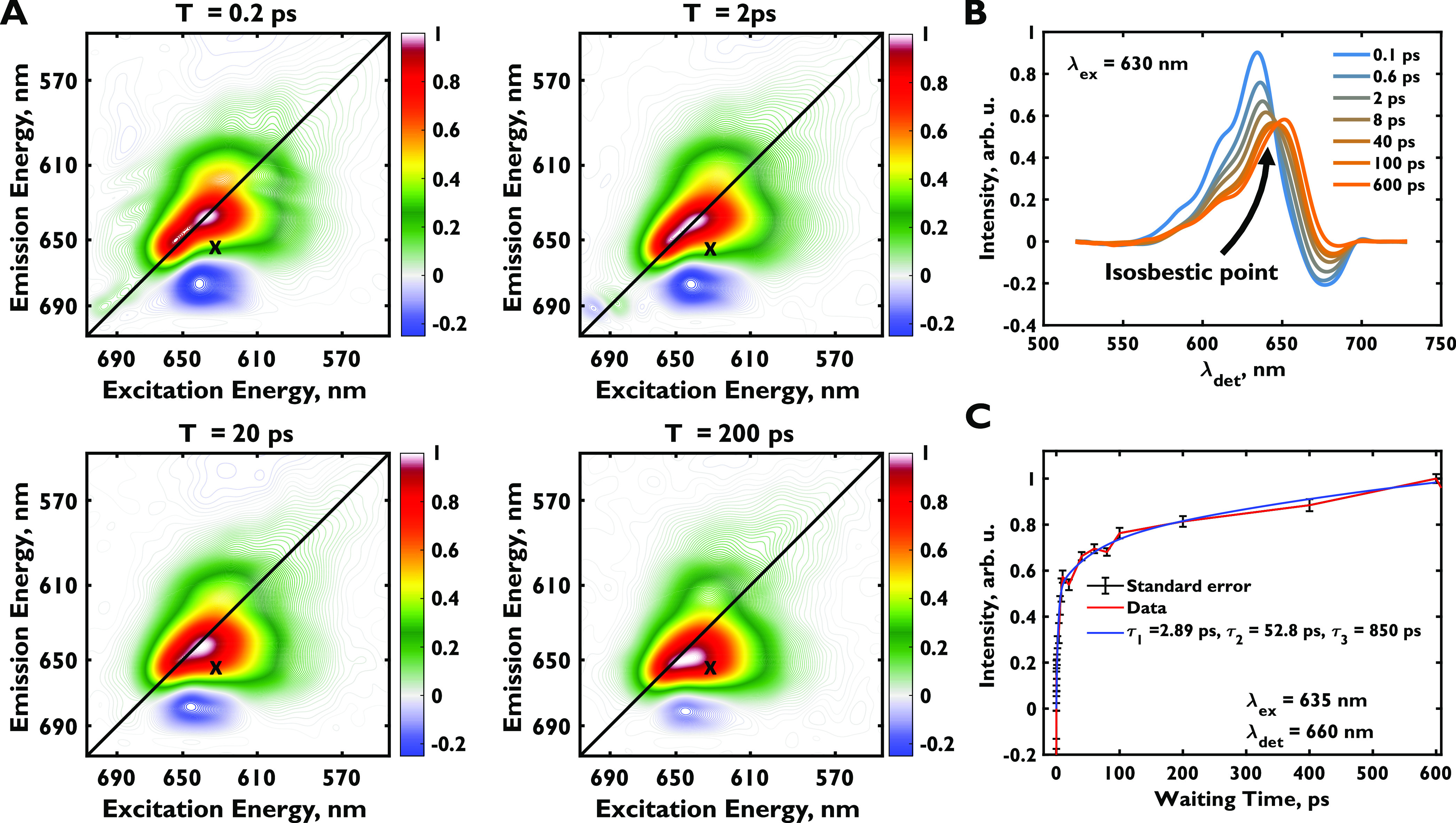
(a) Purely absorptive real-valued two-dimensional electronic spectra of the phycobilisome at 0.2, 2, 20, and 200 ps in the {0, 0, 0, 0°}, or all-parallel pulse sequence. Spectra are frame-normalized. The positive features represent ground-state bleach and stimulated emission, and negative features represent photoinduced absorption. (b) Cross section of the two-dimensional spectra at various waiting times at λ_ex_ = 630 nm. (c) Multiexponential fit for the off-diagonal point, λ_ex_ = 635 nm, λ_det_ = 660 nm.

Two-dimensional spectra of the phycobilisome obtained with identically linearly polarized pulses at 0.2, 2, 20, and 200 ps (Figure 2a) show dynamics in agreement with past measurements and significant spectral congestion.^18−22^ The elongated positive feature along the diagonal from 590 to 670 nm in these spectra at early times corresponds to ground-state bleach and stimulated emission of all chromophores. Phycocyanobilin, the only bilin type in the *Synechocystis* sp. PCC 6803 phycobilisome, has different spectral properties in different protein environments, which gives rise to a broad and elongated lineshape along the diagonal. Each phycocyanin rod hexamer contains six β_155_ bilin chromophores in the β subunit that absorb maximally at 594 nm and six pairs of β_84_ and α_84_ chromophores arranged in close proximity that absorb at 625 and 618 nm, respectively.^42,43^ Each allophycocyanin trimer in the core also contains three β_84_–α_84_ chromophore pairs absorbing maximally at 651 nm.^21^ The maximal emission of the rods is at 645 nm,^44^ and the entire phycobilisome complex emits at ∼665 nm. These features cannot be deconvolved at room temperature because of the broad lineshapes, as seen with other large photosynthetic proteins.^45,46^ Low-temperature studies of the phycobilisome are challenging because of the propensity of the complex to disassemble,^4^ and only a few fluorescence studies have been reported.^47−50^ Similar diagonal elongation in 2D spectra is also observed in other light-harvesting antennae like PSI^45^ and LHCII-CP29-CP24.^46^ The negative feature seen below the diagonal at detection wavelengths (λ_det_) between 660 and 680 nm has previously been attributed to an electrochromic shift of the excited states of neighboring chromophores, which induces a PIA feature.^21^ A rounding out and downward movement in the 610–630 nm excitation region is seen in the positive feature at later times.

Downhill energy transfer from phycocyanin to allophycocyanin should appear as a cross-peak centered at excitation wavelength λ_ex_ = 618–635 nm and λ_det_ = 658 nm, based on chromophore excitation and emission energies. We cannot isolate the rise of this cross-peak because this region is also congested with the decaying PIA feature,^21^ a dynamic Stokes shift in the phycocyanin and allophycocyanin pigments,^51^ and the dynamics of the broad diagonal peak. Figure 2b shows a cross section at λ_ex_ = 630 nm to illustrate this point. Three rising exponentials are needed to accurately fit this region (Figure 2c). The salient isosbestic point at λ_det_ ∼ 647 nm, observed in the cross section in Figure 2b, strongly suggests that a decaying spectral signature is giving rise to a new spectral signature. In previous studies, an ∼50 ps time constant has been attributed to the downward flow of energy from the rods to the core,^19,21^ but this time constant could easily arise from differences between the kinetics of the three processes described above. The first time constant, 2.89 ps, has been attributed to energy transfer along phycocyanin hexamers^19,21^ but could also arise from the large dynamic Stokes shift of 30 nm in phycocyanin rods.^51^ The longest time constant, 850 ps, has been attributed to the fluorescence lifetime and the decay of the PIA feature.^21^ However, spectral congestion from numerous spectroscopic signals prohibits us from attributing these time constants to specific processes.

### Two-Dimensional Electronic Spectroscopy in the Diagonal-Suppressing Pulse Polarization Sequence

Previous studies have used different methods to deconvolve 2DES dynamics in spectrally congested systems including global analysis,^45^ lifetime density analysis,^52^ and cross-peak enhancing pulse sequences.^27^ Polarization control has also been leveraged in many 2DES and 2D infrared spectroscopy studies to extract chiral,^53^ coherence-specific^54,55^ and cross-peak-specific^27,28,56−60^ dynamics. To suppress spectral congestion and selectively watch energy transfer dynamics within the phycobilisome, we used a 2DES pulse sequence that suppresses signals arising from four interactions with parallel transition dipole moments. The suppression is achieved by independently controlling the polarization of all pulses.^59^ The diagonal-suppressing sequence is encoded into the beam polarizations as {90, 60, 120, 0°}^27,28^ or {60, 120, 0, 0°},^59,60^ where the first two pulses are pump pulses, the third is the probe, and the fourth is the local oscillator pulse. Suppressed signals include diagonal signals as well as off-diagonal signals arising from Stokes shifts and ultrafast solvation, as these processes typically do not strongly reorient the transition dipole. Similarly, for linear molecules, ESA signals from the same chromophore are strongly suppressed. We perform 2DES on the phycobilisome complex with both diagonal-suppressing pulse sequences and obtain identical dynamics, which we attribute to energy transfer in the complex. Throughout this work, we refer to diagonal peaks as peaks arising from the same dipole moments as the excitation and cross-peaks as peaks arising from interactions with dipole moments with orientations that differ from those originally excited. Both polarization combinations show identical dynamics because they report on energy transfer between nonparallel stationary dipoles occurring over time.

**Figure 3:**
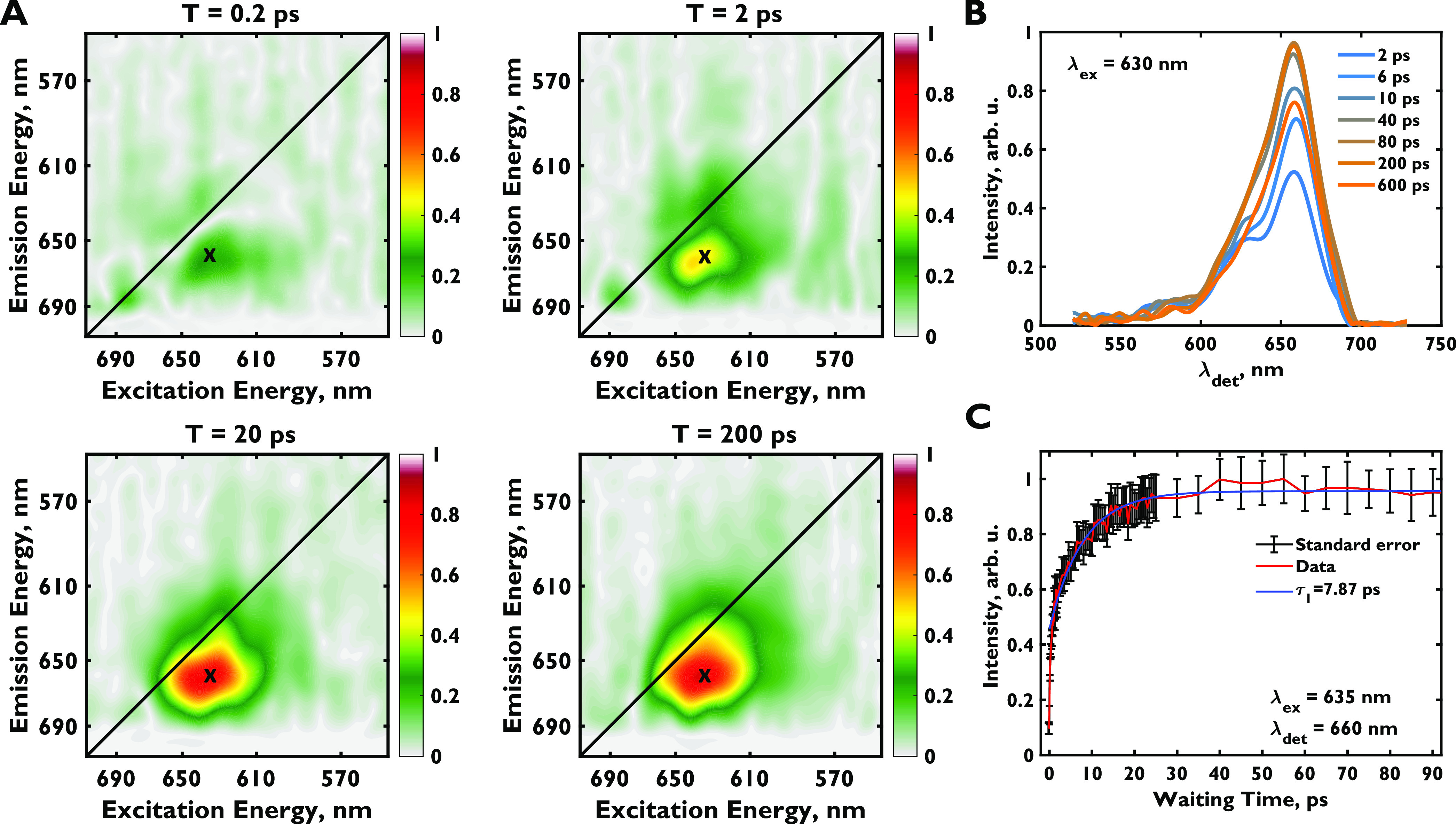
(a) Diagonal suppressed two-dimensional electronic spectra ({90, 60, 120, 0°} pulse sequence) of the phycobilisome at 0.2, 2, 20, and 200 ps. Spectra are normalized to the maximum of the entire data cube. (b) Cross section of the two-dimensional spectra at various waiting times at λ_ex_ = 630 nm. (c) Multiexponential fit for the off-diagonal point, λ_ex_ = 635 nm, λ_det_ = 660 nm. Representative two-dimensional spectra for the {60, 120, 0, 0°} are shown in Supporting Figure 4.

Figure 3a shows two-dimensional spectra in the {90, 60, 120, 0°} pulse sequence at 0.2, 2, 20, and 200 ps. As expected, no signal is seen at early times because of diagonal suppression. At later times, a cross-peak centered at λ_ex_ = ∼631 nm and λ_det_ = ∼657 nm (Figure 3b) rises on the ∼8 ps timescale (Figure 3c). The feature remains stationary and does not arise from reorientation during a dynamics Stokes shift. We attribute this feature to downhill energy transfer from the phycocyanin rods to the allophycocyanin core. The signal decays with a few ns time constant when data is collected up to 800 ps, verifying this assignment. (Supporting Figure 1). This decay is in excellent agreement with the known fluorescence lifetime of the phycobilisome core.^21,22,26^ Our observed timescale of ∼8 ps is closer to the 13 ps timescale suggested by the global analysis of 2DES data by Beck and co-workers^20^ but much faster than timescales obtained from other similar compartmental models.^19,21,22,26^ Moreover, Moran and co-workers do not see a signal redder than λ_det_ = ∼645 nm along the detection axis in photon echo peak shift spectra of C-phycocyanin.^36^ The phycocyanin emission maximum is also at 645 nm.^44^ The signal extends to the blue side of 600 nm in the excitation domain, which is outside of the allophycocyanin absorption, confirming that excitation primarily occurs in the rods. The emission maximum of the peak lies on the emission maximum of the allophycocyanin proteins, ApcA and ApcB. No dynamic Stokes shift is seen even at the earliest times,^51,61^ which would be the case if the signal were due to transfer between C-phycocyanin trimers. Such a signal, which would peak at 645 nm, is clearly seen in the study by Moran and co-workers to occur on the 120 fs timescale.^36^ Finally, our phycobilisome sample emits at λ_max_ = 664 nm in accordance with the literature.^62^ The cross-peak has a λ_max_ of ∼657 nm on the detection axis, which corresponds to emission from the upper rods of the core, in accordance with the emission profile of the quenched phycobilisome in the same work,^62^ further confirming that the primary signal we isolate arises from energy transfer from rods to the first sites in the core. We do not see the small, few nm dynamic Stokes shift of allophycocyanin because this process is fast compared to excitations moving to the core, nor do we see a shifting peak from phycocyanin relaxation because the direction of the dipole moment does not change during this process.

We do not observe signal at early and zero times at the spectral position of the negative signal seen in the all-parallel polarized spectra (Figure 2). We attribute the lack of *T* = 0 signal, which would be indicative of direct coupling between transitions, to two factors: the sparse arrangement of the chromophores leading to relatively localized states and the small fraction of rod chromophores that are coupled to the core. The signal at longer times is effectively amplified because energy absorbed into any rod chromophore eventually traverses from rod to core giving rise to the energy transfer signal. Therefore, the negative signal observed in pump-probe spectra and parallel 2DES is attributed to ESA from S_1_ → S*_n_*, which must have a transition dipole moment nearly parallel to the S_0_ → S_1_ transition. This assumption is reasonable because phycocyanobilin is an approximately linear molecule. It has been recently shown by Beck and co-workers^20^ that excitations in phycocyanin hexamers remain delocalized over the α_84_–β_84_ chromophore pair. The canceling of the ESA feature in our data suggests that in these nearly linear molecules, transition dipoles from singly excited states to higher-lying states are nearly parallel to the transition dipoles from the ground to singly excited states. If the PIA signal were a photoinduced absorption from an electric field-induced shift on a neighboring molecule, this signal would appear from *T* = 0 in the diagonal-suppressing sequence as well. Tensor component analysis by Mukamel and co-workers^63^ confirms that a signal from a nonparallel dipole would not be canceled by our pulse sequence. A similar red ESA feature is observed in an earlier transient absorption study with parallel polarized pump and probe pulses on free phycocyanobilin in solution, which also supports our ESA assignment.^64^ Our data are further supported by a cross-peak observed at λ_ex_ = ∼600 nm and λ_det_ = ∼635 nm, corresponding to energy transfer from the β_155_ chromophore to the β_84_ chromophore and calculated to be 25 ps by Sauer and Scheer (Supporting Figure 2a).^33^ We see a rise of 17 ps (Supporting Figure 2b) and attribute the discrepancy to inter-rod pathways near the core ends^65^ of the rods where β_155_ chromophores are proximal to β_84_ chromophores from other rods. We note that minor contributions from spectral congestion from the broad signal of the main cross-peak likely also influence the time constant.

To confirm that the observed cross-peak, rising on the ∼8 ps timescale, signifies energy transfer from the rods to the core, we revisit the isosbestic point in the fully absorptive, all-parallel polarization sequence 2DES spectra (Figure 2b). Specifically, we look at slices along the detection axis for λ_ex_ = 630 nm. The isosbestic point in these spectra arises from a decay in the spectral signature of the rods and a concomitant rise in the cross-peak of energy transfer from rods to the core. We assume that a dynamic Stokes shift in C-phycocyanin has occurred by 200 fs. At 200 fs, the spectrum is composed entirely of the first spectral signature, the excited state spectrum of the rods and the core. By 600 ps, the spectrum is composed almost entirely of the second component, the cross-peak of rod-to-core energy transfer. Therefore, we fit the spectrum at every time point between 200 fs and 600 ps to a weighted sum of the spectra of the 200 fs signature and the 600 ps signature. These isosbestic fits are found to accurately replicate spectra of all waiting times. We subtract the contribution of the early time spectrum from all time points and plot the resulting spectra. These spectra resemble the cross-sections at λ_ex_ = 630 nm of the cross-peak specific spectra (Supporting Figure 3a,b) and multiexponential fitting of the resulting spectra yields a rise time constant of 7.5 ps (Supporting Figure 3c). This analysis suggests that the rising cross-peak is mostly, if not completely, positive in sign and is hidden below the diagonal and ESA features in the all-parallel data. A rising positive cross-peak is consistent with stimulated emission from the upper core proteins, ApcA and ApcB, after energy transfer from the rods. Singular value decomposition of the purely absorptive parallel polarized 2DES data also yields a cross-peak component that rises with a 6.2 ps time constant (see Supporting Figure 10).

Suppressing the diagonal peaks isolates the intercomplex energy transfer signals from rods to cores and allows us to attribute a timescale to this process. Previous studies have attributed a timescale of 50 ps for downhill energy flow or rod-core equilibration using decay-associated spectra of pump-probe measurements.^15,19,21^ However, we observe a significantly faster rise time of the cross-peak and steady intensity up to ∼150 ps, after which the signal starts to decay. Our obtained dynamics in the all-parallel data acquisition sequence match earlier reports and compartmental models yielding a sub-5 ps component, an ∼50 ps component and an ∼1 ns component. Similar dynamics have been observed in many studies^15,18−23,25,26^ by fitting transient absorption and time-resolved emission decay traces. Our cross-peak specific spectra show distinctly different dynamics from these reports and our own all-parallel 2DES because the pulse sequence selects the cross-peak signal, or the stimulated emission from energy transfer, while suppressing the highly wavelength-dependent dynamics of the other three signatures. In all-parallel 2DES and transient absorption, the time-dependent change of signal intensity is a convolution of the different dynamics of all of these processes making the selective isolation of energy transfer unreliable. The large background of the diagonal signal overwhelms the dynamic response in transient absorption and all-parallel 2DES.

## Discussion

Based on the Förster calculations of Sauer and Scheer,^33^ energy flow along rods of phycocyanin hexamers should occur primarily through intertrimer exciton hopping between adjacent β_84_^1^ and β_84_^4^ rod chromophores. This hop occurs with a FRET rate of 1/(2.5 ps).^18,33^ To rationalize the fast downhill energy transfer rate, we initially used a random hopping model of excitons along four isoenergetic β_84_ sites with a wall on one end and an allophycocyanin sink on the other. Four sites are used based on the negative staining EM images of Gao and co-workers^9^ and other TEM and theoretical studies of phycocyanin assembly and rod-length as functions of light intensity used for cellular growth, which also suggest that two hexamers or four trimers are most commonly found in intact phycobilisome structures.^66,67^ Considering the allophycocyanin as a sink is not easily justified because many studies suggest that the bottleneck for excitation transfer in the phycobilisome is from the rod to the core because of the large interchromophore distance.^8,15^ However, even assuming an allophycocyanin sink, we retrieve only a mean phycocyanin to allophycocyanin transfer time of 16 ps (see the Supporting Information), which does not agree with our measurements.

**Figure 4:**
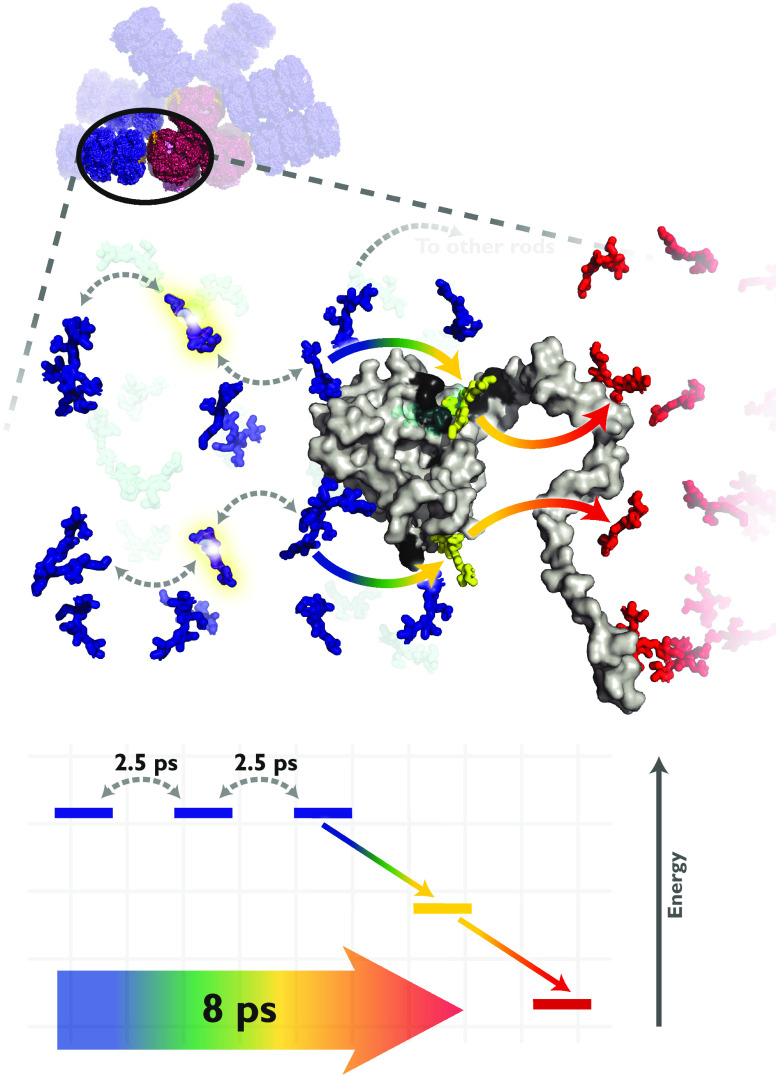
Phycocyanobilin rod chromophores in a single phycobilisome rod (blue) and the nearest allophycocyanin core chromophores (red). Chromophores shown in bold transfer energy directly to the core-proximal chromophores (gold). CpcG, the rod-core linker protein is shown in gray, and its aromatic and charged residues (shown in dark gray) surround the core-proximal rod chromophores (gold). All structures are rendered using coordinates from Kerfeld and co-workers.^15^ Chromophore–residue interactions lower the energy of the core-proximal chromophores by ∼250 cm^–1^ to facilitate unidirectional flow from the rod to the core.^29^

Next, we consider a model in which the last β_84_ chromophore closest to the allophycocyanin core, or the core-proximal β_84_ chromophore, is of lower energy and, as such, an intermediate trap state. Further, the allophycocyanin core is placed at an energy lower than this terminal β_84_ chromophore. In this case, a random walk is allowed between the two most distant chromophores, but excitons effectively move unidirectionally to the core-proximal chromophore from the third chromophore and from this core-proximal β_84_ chromophore to the allophycocyanin core. With this model, we obtain a rod-to-core transfer time of 8.8 ps, in excellent agreement with our measured time constant of 7.9 ps (Supporting Figure 3c). Figure 4 shows a cartoon schematic of this model, which is based on numerous spectroscopic and structural studies: recent cryo-EM structures suggest that aromatic and charged residues of the CpcG rod-core linker protein form a pocket around core-proximal β_84_ chromophores, lowering their energy and forming a conduit through which energy is funneled to the core (Figure 4).^9,15^ Sui and co-workers have previously suggested that this feature is conserved across phycobilisome complexes of cyanobacteria and red algae, and it is also consistent with the spectroscopic characterization of the CpcG-C-phycocyanin linker protein complex.^8,12^ Pertinently, Sauer and Pizarro^29^ and Glazer and co-workers^68^ have characterized the CpcG-phycocyanin complex spectroscopically and a redshift of 12 and 6 nm is seen in the absorption and emission profiles, respectively, of the C-phycocyanin trimer upon CpcG binding. Based on the relative intensity weights of fluorescence peaks, fluorescence spectra of the CpcG-C-phycocyanin complex^29^ suggest that two of the three terminal chromophores red-shift due to interactions with CpcG. The recent cryo-EM structures of the megacomplex suggest that these two chromophores are also the closest contacts to the allophycocyanin core although systematic electronic structure calculations will be needed to obtain the spectra of each chromophore.^9,15^ We also modify our model to incorporate FRET rates based on the absorption and emission spectra of the isolated CpcG-C-phycocyanin complex.^29^ We note however that these spectra contain emission from both red-shifted and non-red-shifted chromophores, which increases back-hopping rates in our calculations and yields an energy transfer time constant of 9.6 ps. Our model incorporates the known redshift of the terminal C-phycocyanin in the FRET rates but is otherwise a simplified FRET model similar to the recent work of Kerfeld and co-workers.^15^ Single-molecule fluorescence studies on this complex may isolate emission from the red-shifted chromophores.^69^ Beck and co-workers have recently shown that excitons hopping along phycocyanin rods are delocalized over the tightly coupled α_84_–β_84_ chromophore pair and that intertrimer transfer predominates localization.^20^ In the case of the core-proximal β_84_ chromophore, the lower energy would likely suppress the superposition of the α_84_–β_84_ states and localize the excitation on the β_84_ chromophore, creating an even more unidirectional flow of energy along the rod.

On average, two β_84_ chromophores of the core-proximal trimer are within 4 nm of the core chromophores. Energy transfer between β_84_ chromophores in the same trimer is slow (1/(25 ps)).^33^ Therefore, an explanation is needed for fast transfer from the third chain of β_84_ chromophores. A few possibilities arise: recent theoretical studies^11,65^ strongly suggest that inter-rod transfer pathways play a dominant role in funneling excitations to the core. Most importantly, the recent down–down and up-down TEM and cryo-EM structures seen by Kerfeld and co-workers^15^ reveal these dominant inter-rod exciton hopping pathways and strongly suggest that excitons are not limited to FRET hopping within single phycocyanin hexamers and rods. From their cryo-EM structures, they calculate that about 70% of phycobilisome complexes have at least one rod in the down conformation. FRET calculations in the same work indicate prominent inter-rod energy transfer pathways. The work by Kerfeld and co-workers^15^ does not suggest that inter-rod transfer significantly speeds up energy transfer to the core because they do not incorporate red-shifted core-proximal chromophores in their modeling. However, up to 10% exciton transfer to each rod from the down-shifted rod is suggested in their calculations.^15^ Another cryo-EM^16^ structure suggests that linker protein interactions laterally shift phycocyanin hexamers with respect to each other along the rod axis. This shift could also lead to more favorable FRET rates.

Finally, the detection maximum of the signal in the cross-peak-specific spectra remains 657 nm until at least 800 ps (Supporting Figure 1). This observation suggests that over the bulk of the exciton lifetime, excitations stay in the APC_660_ pigments. Global analysis on time-resolved fluorescence spectra of the same phycobilisome complex by van Amerongen and co-workers suggests that APC_680_ is reached in 43 ps in rod-less CK phycobilisomes, but this movement was not clearly resolved in wild-type phycobilisomes.^22^ We find that in our wild-type phycobilisomes, bulk excitons from APC_660_ reach the terminal emitters on a much slower timescale. Our observation of the long lifetime of the APC_660_ is also consistent with the study of Beck and co-workers, who suggest that exciton localization may occur in the upper chromophores of the core.^20^ It has been previously shown that the APC_680_ proteins are not involved in OCP-based photoprotection.^48^ The cryo-EM structures of this phycobilisome^15^ show that four OCPs bind to the sides of the core and are suitably placed to quench the majority of the APC_660_ subunits. These observations together suggest that the long staying time of the excitations in the APC_660_ subunits serves as a design strategy to allow efficient OCP-based quenching.

## Conclusions

The phycobilisome antenna differs from the LHCII and LH2 complexes found in plants, algae, and purple phototrophs in that the arrangement of chromophores in the rods and cores is sparse and the chromophores themselves are more fluorescent than chlorophyll derivatives. Unlike other complexes, the phycobilisome does not exploit dense packing of chromophores and quantum mechanical delocalization of excitonic states.^5^ The near-unity efficiency of energy transfer along the phycobilisome is therefore remarkable. What the phycobilin chromophores lack in energy absorption and retention capabilities in comparison to chlorophyll derivatives, they make up for with facile absorption and emission wavelength tunability through their local protein environment, variable conjugation length, and tunable charge-separated states. The known red-shifted core-proximal chromophores increase the probability of excitation energy transfer to the core, reminiscent of similarly placed red chromophores in other systems such as the red chlorophylls in PSI and bacteriochlorophylls in LH1 in purple bacteria.^70,71^ However, in this case, the known red-shifted chromophores are energetic intermediates in relation to the phycobilisome rod and allophycocyanin core chromophores (Figure 4),^29^ so rather than retarding exciton transfer to the next component they improve spectral overlap and speed up energy transfer.

While multiple time-resolved spectroscopic studies have tried to observe site-specific excitation transfer in large and physiologically important light-harvesting antennas, spectral congestion of several antenna components obscures the dynamics. In this study, we suppress diagonal features in 2DES to directly watch exciton flow across tens of nanometers between the components of the phycobilisome megacomplex. The diagonal suppressing pulse sequence suppresses both strong diagonal signal and PIA near the cross-peak, isolating off-diagonal cross-peaks indicative of energy transfer.

Our results show that, despite slow FRET rates within phycocyanin trimers on the order of 10–30 ps and large interchromophore distances, most rod excitations reach the core of the phycobilisome with an ∼8 ps time constant before decaying with a few ns decay constant matching the fluorescence lifetime of the core. Previous studies have pointed out that the rod-to-core transfer is the biggest bottleneck in downhill energy movement in the phycobilisome,^15,72^ but cross-peak specific spectra suggest that this bottleneck is averted by limiting the active random walk region available to the excitation in a rod and by facilitating spectral overlap with the core. Because random walk time scales as N^2^ for N sites in one-dimension (higher for larger dimensions) as assumed for a rod, the rate of transfer doubles when the spectroscopically characterized red-shifted core-proximal chromophore holds excitations to prevent them from escaping back into the random walk region. Reducing the random walk time minimizes the probability of trapping, fluorescence, and exciton annihilation^18,19^ and increases the efficiency of the excitation transfer process. In other words, creating a continuous or fine-tuned spatioenergetic funnel allows largely unidirectional energy flow and enhanced transfer efficiencies. Moreover, inter-rod energy transfer between closely situated chromophores in different rods further lowers reliance on the slow FRET rates within a phycocyanin trimer. Recently observed down–down phycobilisome structures may play a significant role in enhancing this effect.^15^ These interactions are conserved across red algal and cyanobacterial phycobilisomes,^8^ suggesting that they are an evolutionary design feature that drives unidirectional and near-unity efficient energy transfer from phycobilisome rods to cores. Finally, while energy transfers swiftly from rods to the core, transfer from APC_660_ core proteins to the terminal emitters in APC_680_ (ApcD, ApcE, ApcF) is slow and likely allows efficient OCP-based photoprotection. Therefore, the bottleneck in the exciton transfer through the phycobilisome is not the rod-to-core transfer but transfer to the APC_680_ terminal emitters from APC_660_ chromophores. Recent cryo-EM structures of OCP-attached phycobilisome cores from Kerfeld and co-workers^15^ and our observation that excitons spend the bulk of their lifetime in the APC_660_ pigments strongly suggest that the bottleneck of energy transfer to the terminal emitters is not an inefficiency, but a design principle that provides robust and self-contained photoprotection to any protein that should receive excitations from the phycobilisome.

## Supporting Information Available

Spectroscopy, protein, data analysis, and simulation methods; additional data, and alternative scenarios (PDF)

## References

[ref1] Blankenship R. E. Molecular Mechanisms of Photosynthesis, 3rd ed.; Wiley-Blackwell: Hoboken, NJ, 2021.

[ref2] Kasting J. F.; Siefert J. L. Life and the Evolution of Earth’s Atmosphere. Science 2002, 296, 1066–1068. 10.1126/science.1071184. 12004117

[ref3] Schirrmeister B. E.; de Vos J. M.; Antonelli A.; Bagheri H. C. Evolution of Multicellularity Coincided with Increased Diversification of Cyanobacteria and the Great Oxidation Event. Proc. Natl. Acad. Sci. U.S.A. 2013, 110, 1791–1796. 10.1073/pnas.1209927110. 23319632 PMC3562814

[ref4] Green B. R.; Parson W. W. Light-Harvesting Antennas in Photosynthesis; Springer Netherlands: Dordrecht, 2003.

[ref5] Scholes G. D.; Fleming G. R.; Olaya-Castro A.; van Grondelle R. Lessons from Nature about Solar Light Harvesting. Nat. Chem. 2011, 3, 763–774. 10.1038/nchem.1145. 21941248

[ref6] Liu H.; Zhang H.; Niedzwiedzki D. M.; Prado M.; He G.; Gross M. L.; Blankenship R. E. Phycobilisomes Supply Excitations to Both Photosystems in a Megacomplex in Cyanobacteria. Science 2013, 342, 1104–1107. 10.1126/science.1242321. 24288334 PMC3947847

[ref7] Kolodny Y.; Avrahami Y.; Zer H.; Frada M. J.; Paltiel Y.; Keren N. Phycobilisome Light-Harvesting Efficiency in Natural Populations of the Marine Cyanobacteria *Synechococcus* Increases with Depth. Commun. Biol. 2022, 5, 727 10.1038/s42003-022-03677-2. 35869258 PMC9307576

[ref8] Sui S.-F. Structure of Phycobilisomes. Annu. Rev. Biophys. 2021, 50, 53–72. 10.1146/annurev-biophys-062920-063657. 33957054

[ref9] Zheng L.; Zheng Z.; Li X.; Wang G.; Zhang K.; Wei P.; Zhao J.; Gao N. Structural Insight into the Mechanism of Energy Transfer in Cyanobacterial Phycobilisomes. Nat. Commun. 2021, 12, 5497 10.1038/s41467-021-25813-y. 34535665 PMC8448738

[ref10] Harris D.; Bar-Zvi S.; Lahav A.; Goldshmid I.; Adir N. The Structural Basis for the Extraordinary Energy-Transfer Capabilities of the Phycobilisome. Subcell. Biochem. 2018, 87, 57–82. 10.1007/978-981-10-7757-9_3. 29464557

[ref11] Kolodny Y.; Zer H.; Propper M.; Yochelis S.; Paltiel Y.; Keren N. Marine Cyanobacteria Tune Energy Transfer Efficiency in Their Light-Harvesting Antennae by Modifying Pigment Coupling. FEBS J. 2021, 288, 980–994. 10.1111/febs.15371. 32428340

[ref12] Ma J.; You X.; Sun S.; Wang X.; Qin S.; Sui S.-F. Structural Basis of Energy Transfer in *Porphyridium purpureum* Phycobilisome. Nature 2020, 579, 146–151. 10.1038/s41586-020-2020-7. 32076272

[ref13] Marx A.; David L.; Adir N. Piecing Together the Phycobilisome. In The Structural Basis of Biological Energy Generation; Springer Netherlands: Dordrecht, 2014; pp 59–76.

[ref14] Peng P. P.; Dong L. L.; Sun Y. F.; Zeng X. L.; Ding W. L.; Scheer H.; Yang X.; Zhao K. H. The Structure of Allophycocyanin B from *Synechocystis* PCC 6803 Reveals the Structural Basis for the Extreme Redshift of the Terminal Emitter in Phycobilisomes. Acta Crystallogr., Sect. D: Biol. Crystallogr. 2014, 70, 2558–2569. 10.1107/S1399004714015776. 25286841 PMC8494197

[ref15] Domínguez-Martín M. A.; Sauer P. V.; Kirst H.; Sutter M.; Bína D.; Greber B. J.; Nogales E.; Polívka T.; Kerfeld C. A. Structures of a Phycobilisome in Light-Harvesting and Photoprotected States. Nature 2022, 609, 835–845. 10.1038/s41586-022-05156-4. 36045294

[ref16] Kawakami K.; Hamaguchi T.; Hirose Y.; Kosumi D.; Miyata M.; Kamiya N.; Yonekura K. Core and Rod Structures of a Thermophilic Cyanobacterial Light-Harvesting Phycobilisome. Nat. Commun. 2022, 13, 3389 10.1038/s41467-022-30962-9. 35715389 PMC9205905

[ref17] Chang L.; Liu X.; Li Y.; Liu C.-C.; Yang F.; Zhao J.; Sui S.-F. Structural Organization of an Intact Phycobilisome and Its Association with Photosystem II. Cell Res. 2015, 25, 726–737. 10.1038/cr.2015.59. 25998682 PMC4456626

[ref18] Navotnaya P.; Sohoni S.; Lloyd L. T.; Abdulhadi S. M.; Ting P.-C.; Higgins J. S.; Engel G. S. Annihilation of Excess Excitations along Phycocyanin Rods Precedes Downhill Flow to Allophycocyanin Cores in the Phycobilisome of *Synechococcus elongatus* PCC 7942. J. Phys. Chem. B 2022, 126, 23–29. 10.1021/acs.jpcb.1c06509. 34982932 PMC8762654

[ref19] van Stokkum I. H. M.; Gwizdala M.; Tian L.; Snellenburg J. J.; van Grondelle R.; van Amerongen H.; Berera R. A Functional Compartmental Model of the *Synechocystis* PCC 6803 Phycobilisome. Photosynth. Res. 2018, 135, 87–102. 10.1007/s11120-017-0424-5. 28721458 PMC5784004

[ref20] Sil S.; Tilluck R. W.; Mohan T. M. N.; Leslie C. H.; Rose J. B.; Domínguez-Martín M. A.; Lou W.; Kerfeld C. A.; Beck W. F. Excitation Energy Transfer and Vibronic Coherence in Intact Phycobilisomes. Nat. Chem. 2022, 14, 1286–1294. 10.1038/s41557-022-01026-8. 36123451

[ref21] Fălămaş A.; Porav S. A.; Tosa V. Investigations of the Energy Transfer in the Phycobilisome Antenna of *Arthrospira platensis* Using Femtosecond Spectroscopy. Appl. Sci. 2020, 10, 4045 10.3390/app10114045.

[ref22] Tian L.; Gwizdala M.; van Stokkum I. H. M.; Koehorst R. B. M.; Kirilovsky D.; van Amerongen H. Picosecond Kinetics of Light Harvesting and Photoprotective Quenching in Wild-Type and Mutant Phycobilisomes Isolated from the Cyanobacterium *Synechocystis* PCC 6803. Biophys. J. 2012, 102, 1692–1700. 10.1016/j.bpj.2012.03.008. 22500770 PMC3318131

[ref23] Hirota Y.; Serikawa H.; Kawakami K.; Ueno M.; Kamiya N.; Kosumi D. Ultrafast Energy Transfer Dynamics of Phycobilisome from *Thermosynechococcus vulcanus*, as Revealed by ps Fluorescence and fs Pump-Probe Spectroscopies. Photosynth. Res. 2021, 148, 181–190. 10.1007/s11120-021-00844-0. 33997927

[ref24] Nganou C.; David L.; Adir N.; Mkandawire M. Linker Proteins Enable Ultrafast Excitation Energy Transfer in the Phycobilisome Antenna System of *Thermosynechococcus vulcanus*. Photochem. Photobiol. Sci. 2016, 15, 31–44. 10.1039/c5pp00285k. 26537632

[ref25] Gwizdala M.; Berera R.; Kirilovsky D.; van Grondelle R.; Krüger T. P. J. Controlling Light Harvesting with Light. J. Am. Chem. Soc. 2016, 138, 11616–11622. 10.1021/jacs.6b04811. 27546794

[ref26] Tian L.; van Stokkum I. H. M.; Koehorst R. B. M.; Jongerius A.; Kirilovsky D.; van Amerongen H. Site, Rate, and Mechanism of Photoprotective Quenching in Cyanobacteria. J. Am. Chem. Soc. 2011, 133, 18304–18311. 10.1021/ja206414m. 21972788

[ref27] Zanni M. T.; Ge N. H.; Kim Y. S.; Hochstrasser R. M. Two-Dimensional IR Spectroscopy Can Be Designed to Eliminate the Diagonal Peaks and Expose Only the Crosspeaks Needed for Structure Determination. Proc. Natl. Acad. Sci. U.S.A. 2001, 98, 11265–11270. 10.1073/pnas.201412998. 11562493 PMC58718

[ref28] Fidler A. F.; Singh V. P.; Long P. D.; Dahlberg P. D.; Engel G. S. Probing Energy Transfer Events in the Light Harvesting Complex 2 (LH2) of *Rhodobacter sphaeroides* with Two-Dimensional Spectroscopy. J. Chem. Phys. 2013, 139, 155101 10.1063/1.4824637. 24160544 PMC3815049

[ref29] Pizarro S. A.; Sauer K. Spectroscopic Study of the Light-Harvesting Protein C-Phycocyanin Associated with Colorless Linker Peptides. Photochem. Photobiol. 2001, 73, 556–563. 10.1562/0031-8655(2001)0730556ssotlh2.0.co2. 11367580

[ref30] Bryant D. A. The Molecular Biology of Cyanobacteria (Advances in Photosynthesis and Respiration, 1); Springer: Dordrecht, Netherlands, 2006.

[ref31] Flombaum P.; Gallegos J. L.; Gordillo R. A.; Rincón J.; Zabala L. L.; Jiao N.; Karl D. M.; Li W. K. W.; Lomas M. W.; Veneziano D.; Vera C. S.; Vrugt J. A.; Martiny A. C. Present and Future Global Distributions of the Marine Cyanobacteria *Prochlorococcus* and *Synechococcus*. Proc. Natl. Acad. Sci. U.S.A. 2013, 110, 9824–9829. 10.1073/pnas.1307701110. 23703908 PMC3683724

[ref32] Garcia-Pichel F.; Belnap J.; Neuer S.; Schanz F. Estimates of Global Cyanobacterial Biomass and Its Distribution. Algol. Stud. 2003, 109, 213–227. 10.1127/1864-1318/2003/0109-0213.

[ref33] Sauer K.; Scheer H. Excitation Transfer in C-Phycocyanin. Förster Transfer Rate and Exciton Calculations Based on New Crystal Structure Data for C-Phycocyanins from *Agmenellum quadruplicatum* and *Mastigocladus laminosus*. Biochim. Biophys. Acta, Bioenerg. 1988, 936, 157–170. 10.1016/0005-2728(88)90232-0.

[ref34] Riter R. E.; Edington M. D.; Beck W. F. Isolated-Chromophore and Exciton-State Photophysics in C-Phycocyanin Trimers. J. Phys. Chem. B 1997, 101, 2366–2371. 10.1021/jp962609l.

[ref35] Womick J. M.; Moran A. M. Exciton Coherence and Energy Transport in the Light-Harvesting Dimers of Allophycocyanin. J. Phys. Chem. B 2009, 113, 15747–15759. 10.1021/jp907644h. 19894754

[ref36] Womick J. M.; Moran A. M. Nature of Excited States and Relaxation Mechanisms in C-Phycocyanin. J. Phys. Chem. B 2009, 113, 15771–15782. 10.1021/jp908093x. 19902910

[ref37] Matsuura K.; Shimada K. Electrochromic Spectral Band Shift of Carotenoids in the Photosynthetic Membranes of *Rhodospirillum molischianum* and *Rhodospirillum photometricum*. Biochim. Biophys. Acta, Bioenerg. 1993, 1140, 293–296. 10.1016/0005-2728(93)90068-q.

[ref38] Bukartė E.; Paleček D.; Edlund P.; Westenhoff S.; Zigmantas D. Dynamic Band-Shift Signal in Two-Dimensional Electronic Spectroscopy: A Case of Bacterial Reaction Center. J. Chem. Phys. 2021, 154, 115102 10.1063/5.0033805. 33752351

[ref39] Niedzwiedzki D. M.; Bar-Zvi S.; Blankenship R. E.; Adir N. Mapping the Excitation Energy Migration Pathways in Phycobilisomes from the Cyanobacterium *Acaryochloris marina*. Biochim. Biophys. Acta, Bioenerg. 2019, 1860, 286–296. 10.1016/j.bbabio.2019.01.002. 30703363

[ref40] Ho M.-Y.; Niedzwiedzki D. M.; MacGregor-Chatwin C.; Gerstenecker G.; Hunter C. N.; Blankenship R. E.; Bryant D. A. Extensive Remodeling of the Photosynthetic Apparatus Alters Energy Transfer among Photosynthetic Complexes When Cyanobacteria Acclimate to Far-Red Light. Biochim. Biophys. Acta, Bioenerg. 2020, 1861, 148064 10.1016/j.bbabio.2019.148064. 31421078

[ref41] Rast A.; Schaffer M.; Albert S.; Wan W.; Pfeffer S.; Beck F.; Plitzko J. M.; Nickelsen J.; Engel B. D. Biogenic Regions of Cyanobacterial Thylakoids Form Contact Sites with the Plasma Membrane. Nat. Plants 2019, 5, 436–446. 10.1038/s41477-019-0399-7. 30962530

[ref42] Mimuro M.; Füglistaller P.; Rümbeli R.; Zuber H. Functional Assignment of Chromophores and Energy Transfer in C Phycocyanin Isolated from the Thermophilic Cyanobacterium *Mastigocladus laminosus*. Biochim. Biophys. Acta Bioenerg. 1986, 848, 155–166. 10.1016/0005-2728(86)90037-x.

[ref43] Demidov A. A.; Mimuro M. Deconvolution of C-Phycocyanin Beta-84 and Beta-155 Chromophore Absorption and Fluorescence Spectra of Cyanobacterium *Mastigocladus laminosus*. Biophys. J. 1995, 68, 1500–1506. 10.1016/S0006-3495(95)80322-X. 7787035 PMC1282044

[ref44] Sauer K.; Scheer H.; Sauer P. Forster Transfer Calculations Based on Crystal Structure Data from *Agmenellum quadruplicatum* C-Phycocyanin. Photochem. Photobiol. 1987, 46, 427–440. 10.1111/j.1751-1097.1987.tb04790.x.

[ref45] Lee Y.; Gorka M.; Golbeck J. H.; Anna J. M. Ultrafast Energy Transfer Involving the Red Chlorophylls of Cyanobacterial Photosystem I Probed through Two-Dimensional Electronic Spectroscopy. J. Am. Chem. Soc. 2018, 140, 11631–11638. 10.1021/jacs.8b04593. 30133281

[ref46] Do T. N.; Nguyen H. L.; Akhtar P.; Zhong K.; Jansen T. L. C.; Knoester J.; Caffarri S.; Lambrev P. H.; Tan H.-S. Ultrafast Excitation Energy Transfer Dynamics in the LHCII-CP29-CP24 Subdomain of Plant Photosystem II. J. Phys. Chem. Lett. 2022, 13, 4263–4271. 10.1021/acs.jpclett.2c00194. 35522529

[ref47] Calzadilla P. I.; Muzzopappa F.; Sétif P.; Kirilovsky D. Different Roles for ApcD and ApcF in *Synechococcus elongatus* and *Synechocystis* Sp. PCC 6803 Phycobilisomes. Biochim. Biophys. Acta, Bioenerg. 2019, 1860, 488–498. 10.1016/j.bbabio.2019.04.004. 31029593

[ref48] Jallet D.; Gwizdala M.; Kirilovsky D. ApcD, ApcF and ApcE Are Not Required for the Orange Carotenoid Protein Related Phycobilisome Fluorescence Quenching in the Cyanobacterium *Synechocystis* PCC 6803. Biochim. Biophys. Acta, Bioenerg. 2012, 1817, 1418–1427. 10.1016/j.bbabio.2011.11.020. 22172739

[ref49] Kuzminov F. I.; Bolychevtseva Y. V.; Elanskaya I. V.; Karapetyan N. V. Effect of ApcD and ApcF Subunits Depletion on Phycobilisome Fluorescence of the Cyanobacterium *Synechocystis* PCC 6803. J. Photochem. Photobiol. B 2014, 133, 153–160. 10.1016/j.jphotobiol.2014.03.012. 24727864

[ref50] Ashby M. K.; Mullineaux C. W. The Role of ApcD and ApcF in Energy Transfer from PSI and PSII in a Cyanobacterium. Photosynth. Res. 1999, 61, 169–179. 10.1023/a:1006217201666.

[ref51] Petkov B. K.; Gellen T. A.; Farfan C. A.; Carbery W. P.; Hetzler B. E.; Trauner D.; Li X.; Glover W. J.; Ulness D. J.; Turner D. B. Two-Dimensional Electronic Spectroscopy Reveals the Spectral Dynamics of Förster Resonance Energy Transfer. Chem 2019, 5, 2111–2125. 10.1016/j.chempr.2019.05.005.

[ref52] Song Y.; Sechrist R.; Nguyen H. H.; Johnson W.; Abramavicius D.; Redding K. E.; Ogilvie J. P. Excitonic Structure and Charge Separation in the Heliobacterial Reaction Center Probed by Multispectral Multidimensional Spectroscopy. Nat. Commun. 2021, 12, 2801 10.1038/s41467-021-23060-9. 33990569 PMC8121816

[ref53] Lloyd L. T.; Wood R. E.; Mujid F.; Sohoni S.; Ji K. L.; Ting P.-C.; Higgins J. S.; Park J.; Engel G. S. Sub-10 Fs Intervalley Exciton Coupling in Monolayer MoS_2_ Revealed by Helicity-Resolved Two-Dimensional Electronic Spectroscopy. ACS Nano 2021, 15, 10253–10263. 10.1021/acsnano.1c02381. 34096707

[ref54] Schlau-Cohen G. S.; Ishizaki A.; Calhoun T. R.; Ginsberg N. S.; Ballottari M.; Bassi R.; Fleming G. R. Elucidation of the Timescales and Origins of Quantum Electronic Coherence in LHCII. Nat. Chem. 2012, 4, 389–395. 10.1038/nchem.1303. 22522259

[ref55] Thyrhaug E.; Tempelaar R.; Alcocer M. J. P.; Žídek K.; Bína D.; Knoester J.; Jansen T. L. C.; Zigmantas D. Identification and Characterization of Diverse Coherences in the Fenna–Matthews–Olson Complex. Nat. Chem. 2018, 10, 780–786. 10.1038/s41557-018-0060-5. 29785033

[ref56] Mehlenbacher R. D.; McDonough T. J.; Kearns N. M.; Shea M. J.; Joo Y.; Gopalan P.; Arnold M. S.; Zanni M. T. Polarization-Controlled Two-Dimensional White-Light Spectroscopy of Semiconducting Carbon Nanotube Thin Films. J. Phys. Chem. C 2016, 120, 17069–17080. 10.1021/acs.jpcc.6b04961. 27182690

[ref57] Westenhoff S.; Palecek D.; Edlund P.; Smith P.; Zigmantas D. Coherent Picosecond Exciton Dynamics in a Photosynthetic Reaction Center. J. Am. Chem. Soc. 2012, 134, 16484–16487. 10.1021/ja3065478. 23009768

[ref58] Read E. L.; Engel G. S.; Calhoun T. R.; Mancal T.; Ahn T. K.; Blankenship R. E.; Fleming G. R. Cross-Peak-Specific Two-Dimensional Electronic Spectroscopy. Proc. Natl. Acad. Sci. U.S.A. 2007, 104, 14203–14208. 10.1073/pnas.0701201104. 17548830 PMC1964816

[ref59] Tokmakoff A. Orientational Correlation Functions and Polarization Selectivity for Nonlinear Spectroscopy of Isotropic Media. I. Third Order. J. Chem. Phys. 1996, 105, 1–12. 10.1063/1.471856.

[ref60] Farrell K. M.; Yang N.; Zanni M. T. A Polarization Scheme That Resolves Cross-Peaks with Transient Absorption and Eliminates Diagonal Peaks in 2D Spectroscopy. Proc. Natl. Acad. Sci. U.S.A. 2022, 119, e2117398119 10.1073/pnas.2117398119. 35115405 PMC8833161

[ref61] Moya R.; Norris A. C.; Kondo T.; Schlau-Cohen G. S. Observation of Robust Energy Transfer in the Photosynthetic Protein Allophycocyanin Using Single-Molecule Pump-Probe Spectroscopy. Nat. Chem. 2022, 14, 153–159. 10.1038/s41557-021-00841-9. 34992285 PMC9977402

[ref62] Stadnichuk I. N.; Yanyushin M. F.; Maksimov E. G.; Lukashev E. P.; Zharmukhamedov S. K.; Elanskaya I. V.; Paschenko V. Z. Site of Non-Photochemical Quenching of the Phycobilisome by Orange Carotenoid Protein in the Cyanobacterium *Synechocystis* sp. PCC 6803. Biochim. Biophys. Acta, Bioenerg. 2012, 1817, 1436–1445. 10.1016/j.bbabio.2012.03.023. 22483736

[ref63] Dreyer J.; Moran A. M.; Mukamel S. Tensor Components in Three Pulse Vibrational Echoes of a Rigid Dipeptide. Bull. Korean Chem. Soc. 2003, 24, 1091–1096. 10.5012/bkcs.2003.24.8.1091.

[ref64] Bischoff M.; Hermann G.; Rentsch S.; Strehlow D.; Winter S.; Chosrowjan H. Excited-State Processes in Phycocyanobilin Studied by Femtosecond Spectroscopy. J. Phys. Chem. B 2000, 104, 1810–1816. 10.1021/jp992083f.

[ref65] Padyana A. K.; Ramakumar S. Lateral Energy Transfer Model for Adjacent Light-Harvesting Antennae Rods of C-Phycocyanins. Biochim. Biophys. Acta, Bioenerg. 2006, 1757, 161–165. 10.1016/j.bbabio.2006.02.012. 16626627

[ref66] Chenu A.; Keren N.; Paltiel Y.; Nevo R.; Reich Z.; Cao J. Light Adaptation in Phycobilisome Antennas: Influence on the Rod Length and Structural Arrangement. J. Phys. Chem. B 2017, 121, 9196–9202. 10.1021/acs.jpcb.7b07781. 28872312

[ref67] de Lorimier R. M.; Smith R. L.; Stevens S. E. Regulation of Phycobilisome Structure and Gene Expression by Light Intensity. Plant Physiol. 1992, 98, 1003–1010. 10.1104/pp.98.3.1003. 16668720 PMC1080301

[ref68] Lundell D. J.; Williams R. C.; Glazer A. N. Molecular Architecture of a Light-Harvesting Antenna. In Vitro Assembly of the Rod Substructures of *Synechococcus* 6301 Phycobilisomes. J. Biol. Chem. 1981, 256, 3580–3592. 10.1016/s0021-9258(19)69648-1. 6782105

[ref69] Squires A. H.; Moerner W. E. Direct Single-Molecule Measurements of Phycocyanobilin Photophysics in Monomeric C-Phycocyanin. Proc. Natl. Acad. Sci. U.S.A. 2017, 114, 9779–9784. 10.1073/pnas.1705435114. 28847963 PMC5604012

[ref70] van Grondelle R.; Novoderezhkin V. I. Energy Transfer in Photosynthesis: Experimental Insights and Quantitative Models. Phys. Chem. Chem. Phys. 2006, 8, 793–807. 10.1039/b514032c. 16482320

[ref71] Sohail S. H.; Dahlberg P. D.; Allodi M. A.; Massey S. C.; Ting P.-C.; Martin E. C.; Hunter C. N.; Engel G. S. Communication: Broad Manifold of Excitonic States in Light-Harvesting Complex 1 Promotes Efficient Unidirectional Energy Transfer in Vivo. J. Chem. Phys. 2017, 147, 131101 10.1063/1.4999057. 28987085 PMC5848712

[ref72] Adir N.; Bar-Zvi S.; Harris D. The Amazing Phycobilisome. Biochim. Biophys. Acta, Bioenerg. 2020, 1861, 148047 10.1016/j.bbabio.2019.07.002. 31306623

