# Supplementary material for: Phycobilisome’s Exciton Transfer Efficiency Relies on an Energetic Funnel Driven by Chromophore–Linker Protein Interactions
Source: J Am Chem Soc. 2023 May 18;145(21):11659–68. doi: 10.1021/jacs.3c01799 (PMC10236497; doi:10.1021/jacs.3c01799)
Supplement: Supplementary file 1 — ja3c01799_si_001.pdf [file ja3c01799_si_001.pdf]

Supplementary Information for

The Phycobilisome's Exciton Transfer Efficiency Relies on an Energetic Funnel Driven by Chromophore-Linker Protein Interactions

Siddhartha Sohoni[1], Lawson T. Lloyd[1], Andrew Hitchcock[2], Craig MacGregor-Chatwin[2, 3], Ainsley Iwanicki[1], Indranil Ghosh[1], Qijie Shen[1], C. Neil Hunter[2], Gregory S. Engel[1]*

1. Department of Chemistry, James Franck Institute and Institute for Biophysical Dynamics, Pritzker School of Molecular Engineering, The University of Chicago, Chicago, Illinois 60637, United States

2. School of Biosciences, University of Sheffield, Sheffield, S10 2TN, UK

3. Current affiliation: Electron Bio-imaging Centre, Diamond Light Source, Didcot, OX11 0DE, UK

*Corresponding author: gsengel@uchicago.edu

Temporal Dynamics of Energy Transfer Features

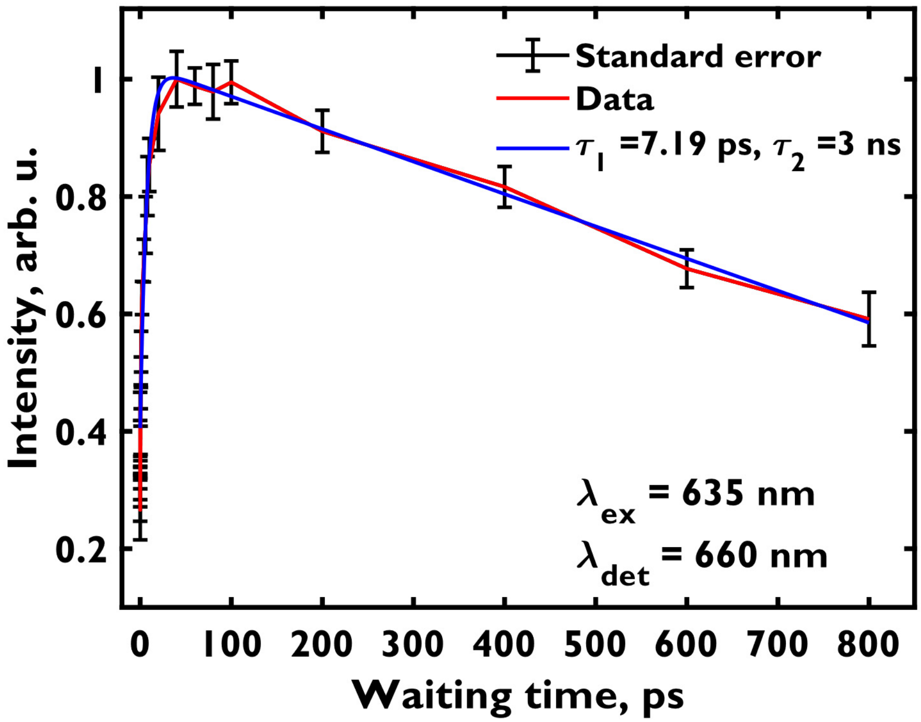

Supplementary Figure 1: Multiexponential fitting for the cross-peak 2DES signal in the {90 º 60 º 120 º 0 º} sequence. The second time constant is ~3 ns, which matches with the fluorescence lifetime of the phycobilisome.

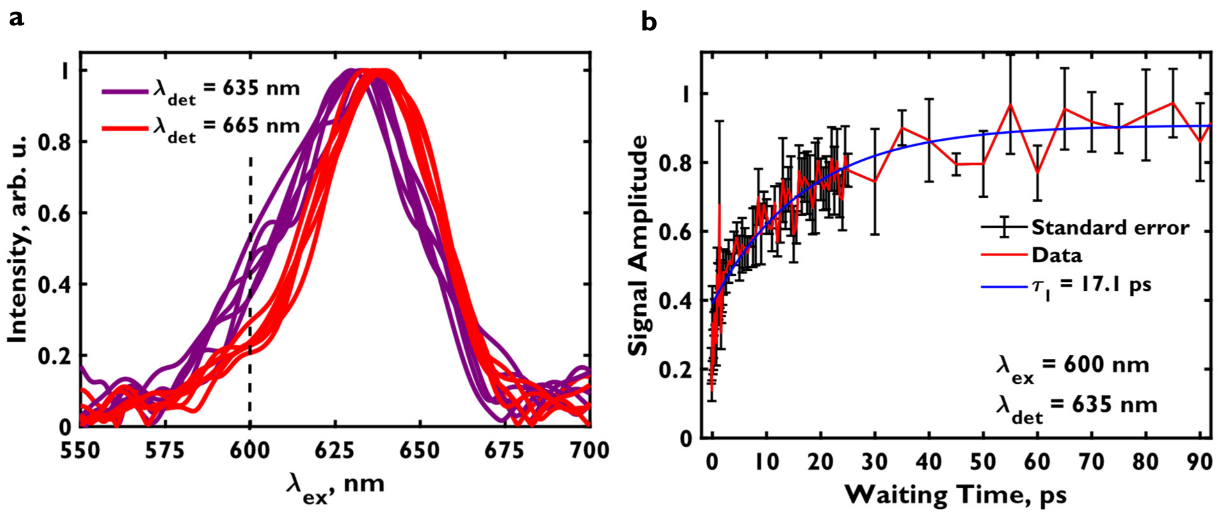

Supplementary Figure 2: a) Normalized cross-sections along excitation axis at detection wavelengths, 635 nm and 665 nm for various waiting times show a peak at λ_{ex} = 600 nm corresponding to energy transfer from the β_{155} chromophore to the β_{84} chromophore **b)** Dynamics of the signal rise at λ_{ex} = 600 nm and λ_{det} = 635 nm.

Isosbestic point analysis:

Pseudo-TA spectra are taken from the purely absorptive 2D spectra for λ_{ex} = 630 nm. Each pseudo-TA spectrum between 0.2 ps and 600 ps is deconvolved into different fractions of the spectrum at 0.2 ps and 600 ps. An example is shown in the bottom right of Supplementary Figure 3 where the spectrum at 20 ps is matched to a linear combination of the spectra at 0.2 ps and 600 ps.

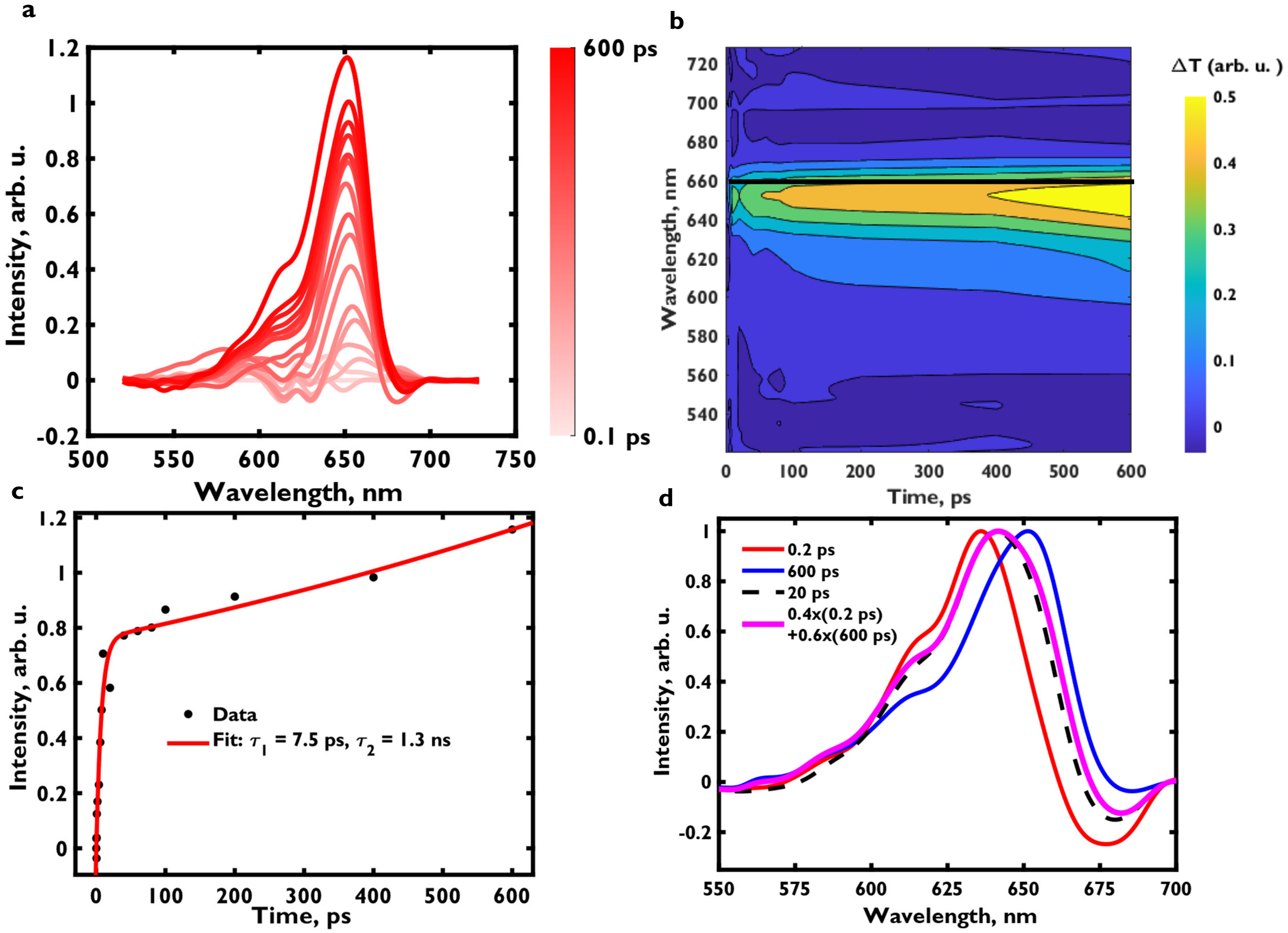

Supplementary Figure 3: Isobestic analysis of the phased parallel polarization 2DES. **a)** Cross-sections at λ_{ex} = 630 nm along the detection wavelength after subtraction of the weighted 200 fs spectrum. This spectrum resembles the cross-peak specific spectra but small inaccuracies in subtraction around the ESA peak (660 nm) show this peak to be blue-shifted by a few nm. **b**) Pseudo-transient absorption waterfall plot for λ_{ex} = 630 nm after subtraction of a weighted 200 fs spectrum from each time-point. **c)** Multi-exponential fitting at λ_{det} = 660 nm for the early-time subtracted spectra shown in part a. A 7.5 ps time constant is seen that corresponds to rise of the cross-peak. **d)** Linear decomposition of the spectrum at 20 ps with the spectra at 0.2 ps and 600 ps.

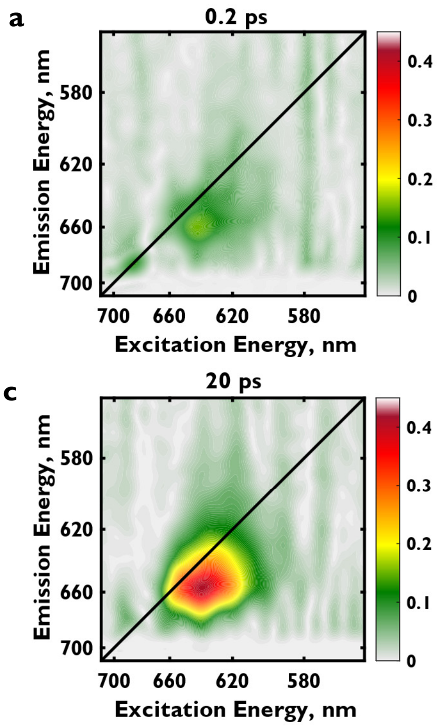

Supplementary Figure 4: Representative 2D spectra for the {60 ° 120 ° 0 ° 0 °} polarization sequence.

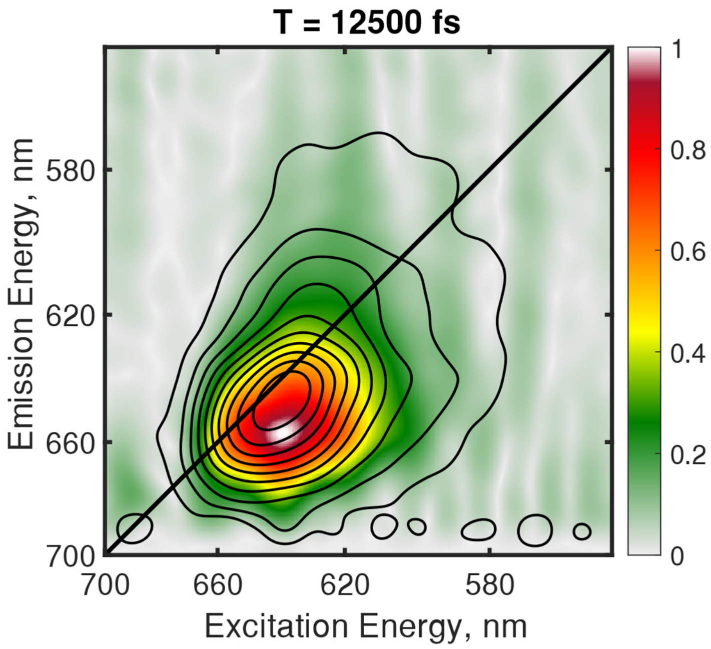

Supplementary Figure 5: Absolute valued parallel polarized 2D spectrum at 12.5 ps (in black lines) overlaid on the cross-peak enhanced 2D spectrum at 12.5 ps to show that the peak positions are different.

Experimental Methods:

UV-vis Absorption Spectroscopy: Absorption spectra are collected in a 1 mm vial in a Cary 5000 spectrometer. Spectra are collected before and after time-resolved data acquisition.

Fluorescence Spectroscopy: Fluorescence spectra are collected on a Horiba JobinYvon spectrometer with an excitation wavelength of 590 nm, entrance and exit slits of 3 nm and an integration time of 0.1 s.

Our all-parallel 2DES data faithfully retrieves the time constant of the APC emission rise of 1 ns across many individual data runs suggesting energy transfer from rods to core and sample intactness.

Two-dimensional Electronic Spectroscopy and Pump-Probe Spectroscopy: Two-dimensional spectroscopic experiments were performed using a setup described previously (ref 53 in main text). Briefly, a Ti:sapphire mode-locked oscillator (Coherent Micra) operating at 80 MHz seeds a Ti:sapphire regenerative amplifier (Coherent Legend Elite) to produce 35 fs pulses centered at 800 nm at a 5 kHz repetition rate. This output is passed through a pressurized argon tube held at ~16 pounds per square inch above atmosphere to generate a white light continuum. The 800 nm light remaining after white light generation is rejected with a bandpass filter, and the white light is compressed using two pairs of chirped mirrors (Laser Quantum) to ~8 fs fwhm. 2D spectra are collected in the fully non-collinear BOXCARS geometry with a beam diameter of 290 μm at the focus at the sample and heterodyned with an attenuated local oscillator pulse. Powers are kept to 15 nJ per pulse in our experiments. At this fluence, annihilation is present but minimal based on the biexponential fitting of Ref. 18 for a fluence corresponding to 2.5 excitations per rod (refs 18-20 of main text). Since that study and other studies have previously proved that annihilation within rods is faster than energy transfer to the core (~2.5 ps), annihilation most likely does not impact the kinetics of the cross-peak signal rise. We do not observe a fast decay constant or nonlinear decays for the cross-peak suggesting annihilation does not occur in the core. Broadband half-waveplates (Union Optic) are used to independently control the polarization of each pulse. Two-dimensional spectra were obtained by collecting coherence time data from -90 to 90 fs in 1.5 fs time-steps. Hann and Tukey windows were used for apodization and windowing in rephasing time and coherence time respectively. Fully-absorptive 2D spectra were obtained for the parallel polarization sequence using the projection-slice theorem after acquisition of separate pump-probe spectra at identical waiting times to determine the absolute signal phase for every waiting time frame independently. A 200 μm flow cell was used and the sample was flowed throughout data acquisition.

Phycobilisome isolation: *Synechocystis* cells were pelleted (14,334 xg, 10 min, 4 °C) and resuspended in a small volume of 25 mM potassium phosphate buffer pH 7.4 containing 100 mM NaCl and 10 mM $MgCl_2$ (approximately 1 liter of cells was resuspended in 10 ml of buffer). The resuspended cells were mixed with an equal volume of 0.1 mm glass beads (Thistle Scientific) and were then broken by 8 rounds of 60 seconds of bead beading with cooling on ice for 3 min between cycles using a Mini-Beadbeater (BioSpec Products). The cell lysate was centrifuged at 100000 x g at 4 °C for 4 hours to pellet glass beads, unbroken cells and cell membranes. The phycobilisome-containing supernatant was then decanted, flash frozen in liquid N_2 and stored at -80 °C.

We note that we initially attempted to perform our measurements on a *Synechococcus* sample that was further purified with a sucrose gradient but found that the increased scatter (by an order of magnitude) from the high-sucrose concentration prevented the collection of statistically significant cross-peak specific signal. This signal is by itself weaker than the diagonal signal in the parallel polarization sequence, and it is further suppressed by a factor of 10 in the cross-peak specific sequences (refs 63, 27 main text) making it difficult to isolate it from the scatter for fluences in which annihilation in minimum (ref 18-20 main text). Our sample quantity did not allow further dilution or dialysis after the sucrose gradient. The fluorescence spectrum of the supernatant does not show any phycocyanin fluorescence confirming the absence of detached distant phycocyanin trimers (ref 9 main text). Detached rods do not contribute to the cross-peak specific signal and detachment does not influence energy transfer dynamics other than possibly speeding up the transfer through inter-rod processes further, if all rods were present on the phycobilisome and inter-rod transfer was enhanced.

Exciton Hopping Simulations:

A simple probabilistic transfer matrix-based exciton hopping model is used to simulate the observed energy transfer dynamics in PBS rods. The position of the exciton is recorded by a 5x1 column vector, $\boldsymbol{x_n}$. The first three entries represent phycocyanin sites, the fourth row represents the terminal phycocyanin and the fifth row represents the allophycocyanin core. The transfer matrix equation of propagation used is $\boldsymbol{Tx_n = x_{n+1}}$. The transfer matrix is a 5x5 matrix whose diagonal elements are zero, and the following elements are non-zero probabilistically:

Scenario 1

T(2, 1) = 1 with a probability of 1 indicating that the exciton always hops from the terminal chromophore to the next one. Between T(3, 2) and T(1, 2) one of the elements is 1 and the other is zero so that the exciton randomly hops to one of its neighbours. The probability of either being 1 is made 50% using a pseudorandom variable with the randi command of Matlab. T(4, 3) and T(5, 4) are set to 1. All other elements are zero. We average 100000 scenarios for each of the following cases: when $\boldsymbol{x_1}$(1, 1) = 1, $\boldsymbol{x_1}$(2, 1) = 1, $\boldsymbol{x_1}$(3, 1) = 1 and $\boldsymbol{x_1}$(4, 1) = 1. The simulation ends when $\boldsymbol{x_n}$(5, 1) becomes 1 because it signifies the exciton reaching the core. Each iteration of the transfer matrix propagation is counted as 2.5 ps. The 2.5 ps exciton hopping time between adjacent trimer β_{84} chromophores is obtained from experiments and theory from the works of Scheer and coworkers (ref 33 in main text) and Engel and coworkers (ref 18 in main text). This model yields an average transfer time constant of 8.75 ps.

An example transfer matrix operating on an $\boldsymbol{x}$ matrix is shown below:

$$\begin{pmatrix} 0 & 0 & 0 & 0 & 0 \\ 1 & 0 & 0 & 0 & 0 \\ 0 & 1 & 0 & 0 & 0 \\ 0 & 0 & 1 & 0 & 0 \\ 0 & 0 & 0 & 1 & 0 \end{pmatrix} \cdot \begin{pmatrix} 0 \\ 0 \\ 1 \\ 0 \\ 0 \end{pmatrix} = \begin{pmatrix} 0 \\ 0 \\ 0 \\ 1 \\ 0 \end{pmatrix}$$

Scenario 2

Next we consider the above model but now assume that the terminal phycocyanin is not an energetic trap, so that energy can transfer back from the third site to the second site instead of unidirectionally moving to the fourth site. We model this scenario using the following change: between T(4, 3) and T(2, 3), one of the elements is one and the other is zero and both scenarios have a probability of 0.5. T(5, 4) = 1 with a probability of 1. We average 100000 scenarios for each of the following cases: when $\boldsymbol{x_1}(1, 1) = 1$, $\boldsymbol{x_1}(2, 1) = 1$, $\boldsymbol{x_1}(3, 1) = 1$ and $\boldsymbol{x_1}(4, 1) = 1$. The simulation ends when $\boldsymbol{x_n}(5, 1)$ becomes 1 because it signifies the exciton reaching the core. As before, each iteration of the transfer matrix propagation is counted as 2.5 ps. This model yields a transfer time of 16.3 ps. If we do not consider the core to be a trap and allow back transfer even from the terminal phycocyanin, which is a more realistic physical possibility owing to the large distance between the terminal phycocyanin and the core allophycocyanin chromophore, we model this scenario with having a 0.5 probability of T(5, 4) = 1 and T(3, 4) = 0 and a 0.5 probability of having T(5, 4) = 0 and T(3, 4) = 1. This model yields a time constant of 31.3 ps. Clearly, these models do not match with the experimentally obtained rise time, strongly suggesting a cascaded energetic landscape.

Scenario 3

We use the spectroscopic characterization of the CpcG-phycocyanin complex conducted by Sauer and Pizarro (ref 29 in main text) to calculate the FRET rate and probability of exciton hopping from the third phycocyanin to the terminal phycocyanin and the back-hopping by the following formula: $T = 2.5*\sigma_{ji}/*\sigma_{ij}$ ps. We get a hopping rate constant of ~1 ps from the third chromophore to the terminal CpcG linked chromophore and a 4 ps time constant for the reverse hopping. From this model, we construct a scenario in the transfer matrix where T(4, 3) = 1 and T(2, 3) = 0 with a probability of 0.8 and T(4, 3) = 0 and T(2, 3) = 1 with a probability of 0.2. This model yields a time constant of 9.6 ps. However, we note that the reverse hopping rate of 1 ps is obtained largely due to spectral overlap between the β_{84} chromophore in the CpcG-PC complex that is not redshifted and the chromophores of the CpcL-PC complex. In the actual system, this chromophore does not transfer excitons to the core directly, so the actual back hopping rate may be negligible due to extremely small spectral overlap. Individual fluorescence of the CpcG surrounded chromophores cannot be separated without single molecule spectroscopy measurements. Similarly, negligible change in spectral overlap is seen when comparing the CpcG-PC complex with the free PC complex as donor and allophycocyanin as acceptor. Here again, the broad blue fluorescence from chromophores not involved in direct energy transfer to the chromophore plays a significant role in dictating the overlap. Selectively observing fluorescence profiles of CpcG interacting chromophores will again need single molecule measurements.

We note here that the CpcL-PC and CpcG-PC fluorescence and absorption spectra used to calculate relative FRET hopping rates are taken from the work of Sauer and Pizarro (ref 29 main text) and they isolated the complexes from *Synechococcus* sp. PCC 7002. The allophycocyanin absorption and fluorescence spectra were obtained from the work of Tosa and coworkers (ref 21 main text) and they isolated these hexamer complexes from *Arthrospira platensis*, but the spectra match those of the *Synechocystis* sp. PCC 6803 complex. Any small changes between species would have negligible impact on the spectral overlap and hopping time. The hopping time between adjacent chromophores of 2.5 ps is obtained from the calculations of Sauer and Scheer (ref 33 main text) which were performed on crystal structures from *Mastigocladus laminosus* and *Agmenellum quadruplicatum*. The hopping time is corroborated with experimentally observed hopping time seen by Engel and coworkers (ref 18 main text) which was measured in *Synechococcus elongatus* PCC 7942. We rely on the cryo-EM structure of CpcG interactions with terminal phycocyanin chromophores mainly from the work on the *Synechocystis* sp. PCC 6803 complex by Kerfeld and coworkers (ref 15 main text). Similar interactions are seen in the study by Gao and coworkers (ref 9 main text) for *Synechocystis* sp. PCC 7002 and in red-algal phycobilisomes (ref 8, 12 main text) by Sui and coworkers. Low fluorescence efficiencies are also quoted for different species, not *Synechocystis* sp. PCC 6803. We note that the full-model of the up-up structure provided by Kerfeld and co-workers is assembled (from EM structures of individual components) and therefore the rod-core chromophore distances are approximate. These distances however closely match those from the structure from Gao and co-workers. Therefore, we reiterate the point that, despite the approximations built into the hopping model, we can show that by incorporating only redshifted chromophores we can replicate the experimentally observed rate. Single-molecule fluorescence studies of the CpcG-PC linker protein complex are needed to obtain the spectral signatures of the redshifted chromophores selectively and to incorporate them into the FRET model.

Verification of Accuracy of Model by Comparison with other FRET models

To verify that our exciton hopping model is consistent with molecular-level cryo-EM structure based hopping models, we compare our model with the model of Kerfeld and coworkers (Ref. 15, main text). To do so, we initialize our model with the same initial conditions as their model. We use the 2.5 ps exciton hopping rate as the average rate based on the structure-based calculations of Sauer and Scheer (Ref. 33 main text). We model the rod with six consecutive sites and the core APC chromophore as the final, seventh site. We do not assume that energy transfer to the core-proximal chromophore is unidirectional (do not incorporate redshifts) and we initialize our simulations such that the excitation is always placed at the very end of the rod (first site). For these initial conditions, we obtain an energy transfer rate of 30 ps from the rods to the core. This rate is in close agreement with the model built by Kerfeld and coworkers (40 ps). We note that while their model shows dynamics for the specific scenario mentioned above, our initialization conditions to explain the faster rate are based on two statistical considerations: 1. We place the excitations at random on any site in the rod. 2. We use four sites instead of six based on statistics observed in a few TEM and nsEM studies. While our initial parameters inherently yield faster transfer rates, we can conclude that our mechanics of propagation of the transfer matrix agree with the detailed, structure-based model of Kerfeld and coworkers.

Singular Value Decomposition of Parallel Polarized, Purely Absorptive 2DES Spectra

SVD is performed on the long-time dataset of the phased (real valued, purely absorptive) all-parallel polarized data to spectrally isolate the cross-peak corresponding to energy transfer. Negative and earliest waiting times (up to 400 fs) are not analyzed to remove effects of pulse-misordering and coherent response that would otherwise contribute to the analysis.

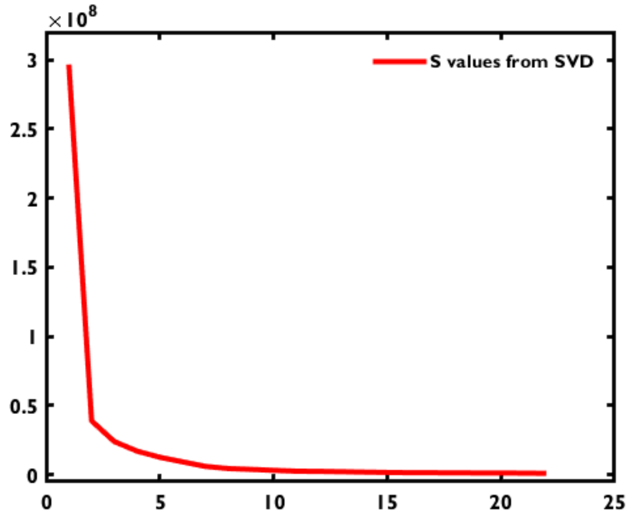

Supplementary Figure 6: Weight of S values upon singular value decomposition of the purely absorptive all-parallel polarized 2DES data cube.

Based on the significances, we look at the first two components and their time-evolution. We present 2D maps of both the SVD components and their associated kinetics recovered from the SVD analysis. Positive signs correspond to GSB and SE and negative signs are photoinduced absorption.

SVD Component 1:

The first component consists of largely the early time spectrum. Its time evolution follows a triexponential decay. The values are similar to the triexponential obtained from fitting a single point in these spectra:

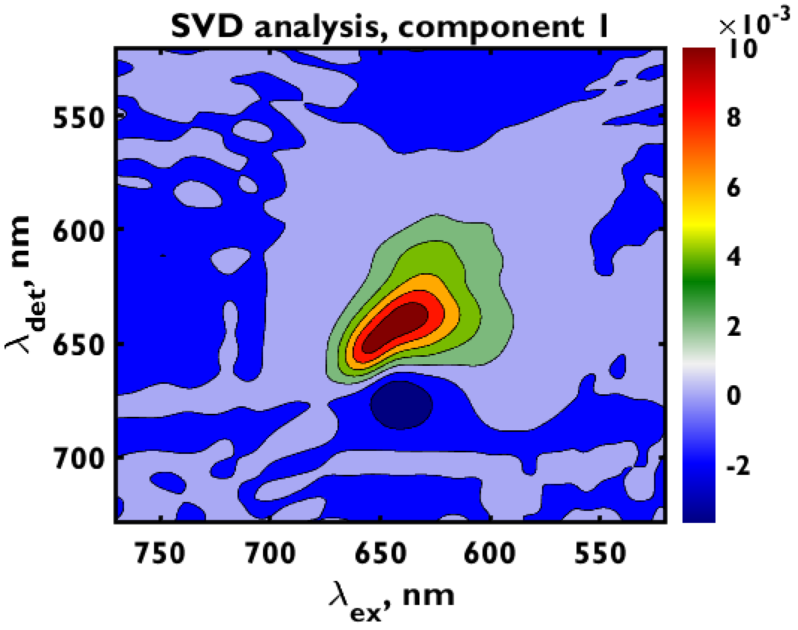

Supplementary Figure 7: The first spectral component (V matrix) from SVD analysis of the purely absorptive all-parallel polarized 2DES data cube. It largely resembles the early time 2D spectrum.

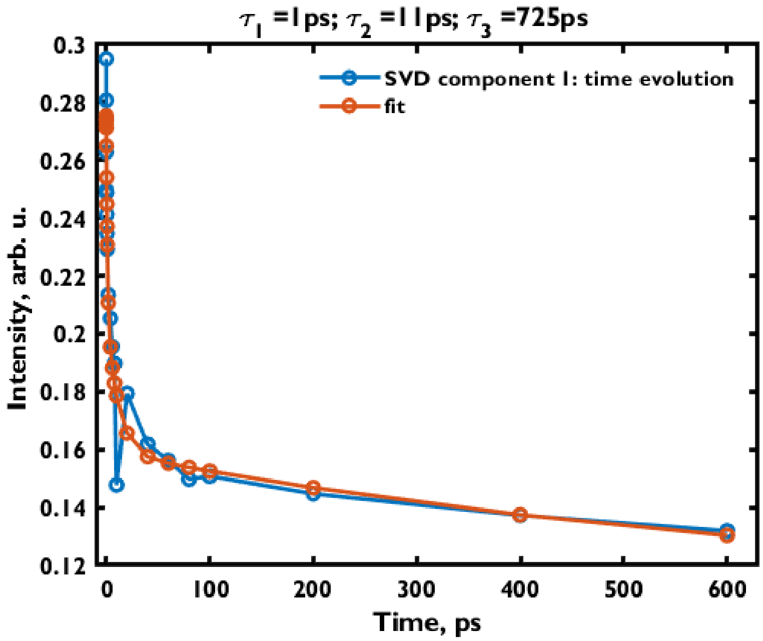

Supplementary Figure 8: Dynamics associated (U matrix) with the first spectral component from the SVD analysis. A multiexponential decay is needed to fit these dynamics.

SVD Component 2

Component 2 does show a cross-peak of positive sign and a corresponding negative intensity on the diagonal indicative of energy transfer. The cross-peak is centered at λ_{ex} = 630 nm and λ_{det} = 660 nm, which would correspond to energy transfer from the rods to the cores. This cross-peak shows a rise time of 6.2 ps and a decay of 911 ps. The rise time is in good agreement with the cross-peak 2DES data.

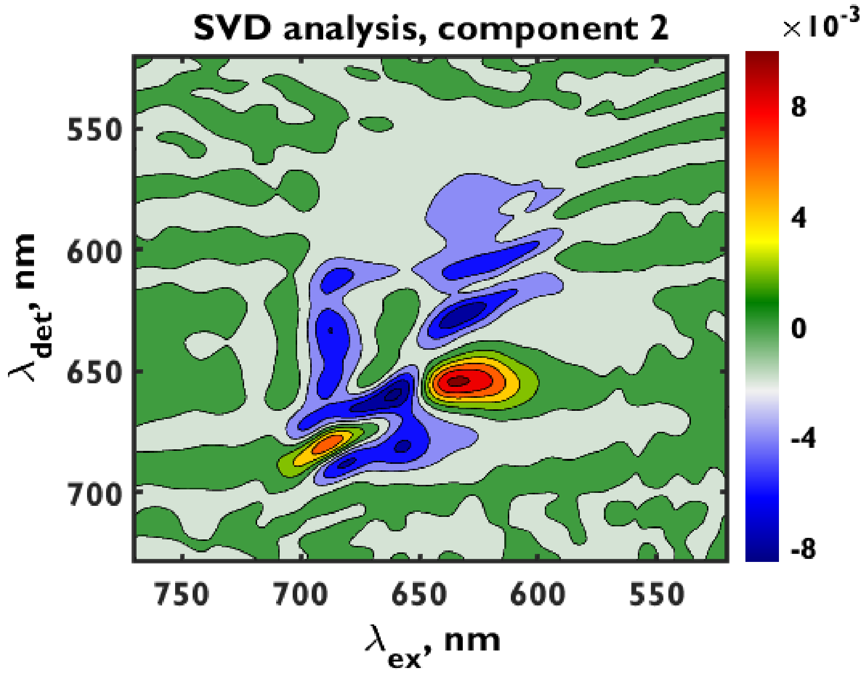

Supp. Figure 9: The second spectral component (V matrix) from SVD analysis of the purely absorptive all-parallel polarized 2DES data cube. It shows a prominent cross-peak at the energy transfer position.

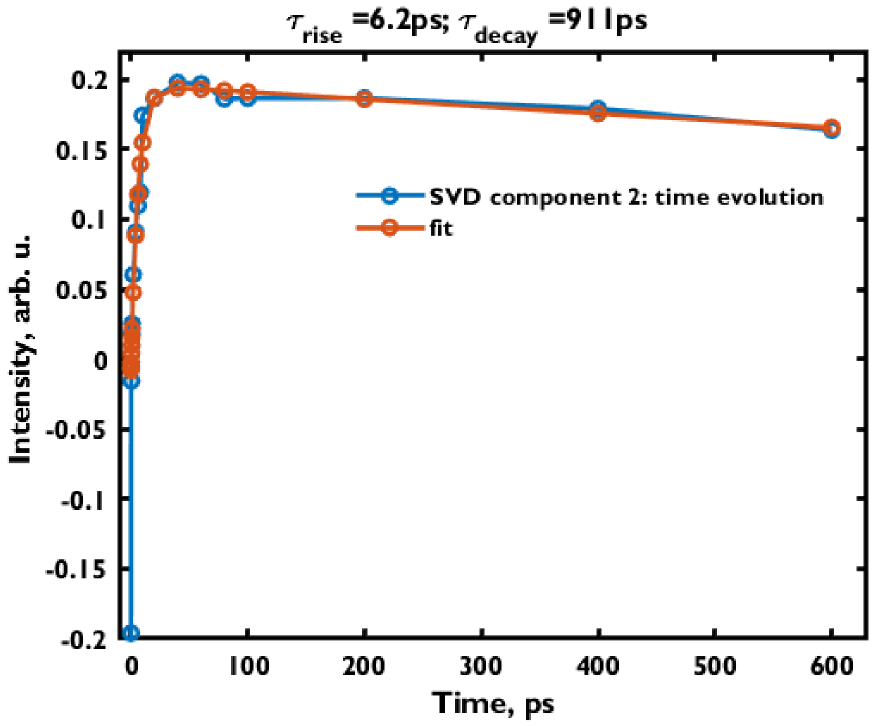

Supp. Figure 10: Dynamics associated (U matrix) with the second spectral component from the SVD analysis. A rise time of ~6 ps is obtained. In performing a biexponential fit to this profile, the large ns time regime background is ignored because of the slow decay of this U component.

Signal Suppression Calculation

We picked one rod in the cryo-EM structure of Gao and co-workers (ref 9, main text) and calculated the dipole moments of the chromophores through singular value decomposition. We found a relative orientation of 52° between the terminal phycocyanin chromophore and the closest allophycocyanin contact. The relative angle between the dipole moments of the terminal rod chromophore and the one before it is 30° and the angle between the terminal chromophore and two chromophores next to it in the same rod is 23°. The SVD results of the vector directions in which the atoms are oriented are listed below:

Closest APC chromophore: [0.5177 0.7310 -0.4445]

Terminal (fourth in rod) PC chromophore: [0.7993 -0.0939 -0.5936]

Third PC chromophore: [0.6527 -0.5869 -0.4791]

Second PC chromophore: [-0.8632 -0.2651 0.4296]

First PC chromophore: [0.3449 -0.8111 0.4724]

The strength of the cross-peak specific signal is a function of the angle between the transferring dipoles, and functionally changes with it as $sin^2\theta$ (Mukamel and coworkers, ref 63 main text). Therefore, the signal from rod-to-core transfer is about two times stronger in our pulse sequence as compared to the signal from transfer within rods.

Alternative Scenarios

In this work, we assign the main cross-peak at λ_{ex} = 630 nm and λ_{det} = 660 nm to energy transfer from rods of the phycobilisome to core. We briefly consider an alternate scenario:

If the limiting step of exciton flow from the rods to the terminal phycobilisome emitter was the step of transfer from the terminal rod phycocyanin chromophore to the core, and equilibration along the rod was much faster than this step, we would see a weak diagonal feature (weak due to the suppression) at ~625-640nm appearing with a fast rise time which would gradually disappear with the time constant of the rise of the below-diagonal peak resulting from rod-to-core transfer. We do not see such a process due to the unidirectionality of the transfer to the core. Since the rise of the peak at the diagonal and below the diagonal is concomitant, we can conclude that hopping along rods unidirectionally leads to rod-to-core transfer (see **Supplementary Figure 12**). The signal on the diagonal decays with the same timescale as the peak below the diagonal due to two reasons: some excitations may not efficiently transfer to the core, and the broad lineshape of the below-diagonal signal provides some contribution to the point at λ_{ex} = 630 nm and λ_{det} = 630 nm.

The presence of this cross-peak as a separate signal is very strongly indicated by both our isosbestic point analysis and the singular value decomposition of the all-parallel polarized data. Both methods yield similar time-constants for the cross-peak growth. We suggest that this strongest cross-peak is the result of downhill energy flow, and not of a dynamic Stokes shift or back-transfer because the fast Stokes shift occurs within 120 fs as is shown by Moran and coworkers (refs 35, 36, main text) whose experiments on both PC and APC serve as controls to our data. From their data, we remind the reader that PC does not show appreciable

signal below 645 nm on the detection axis and APC has a signal maximum at ~660 nm on both axes. A back-transfer process would yield a peak on the diagonal or slightly above it.

Additional Spectra and Long Time Dynamics:

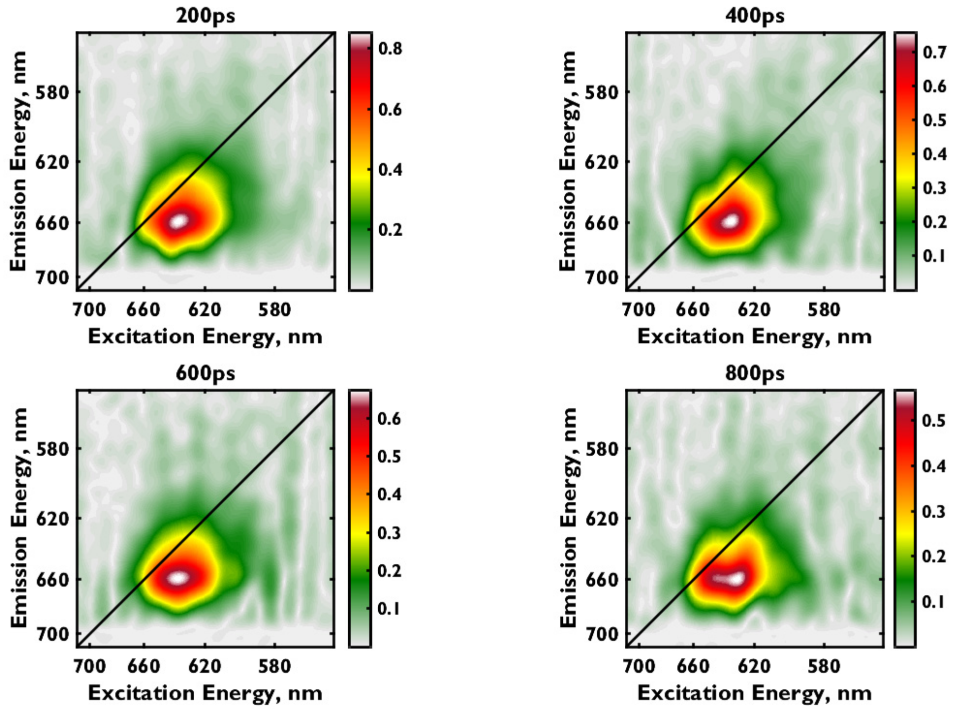

Supplementary Figure 11: Cross-peak polarized spectra from the {90 º 60 º 120 º 0 º} sequence at 200, 400, 600 and 800 ps.

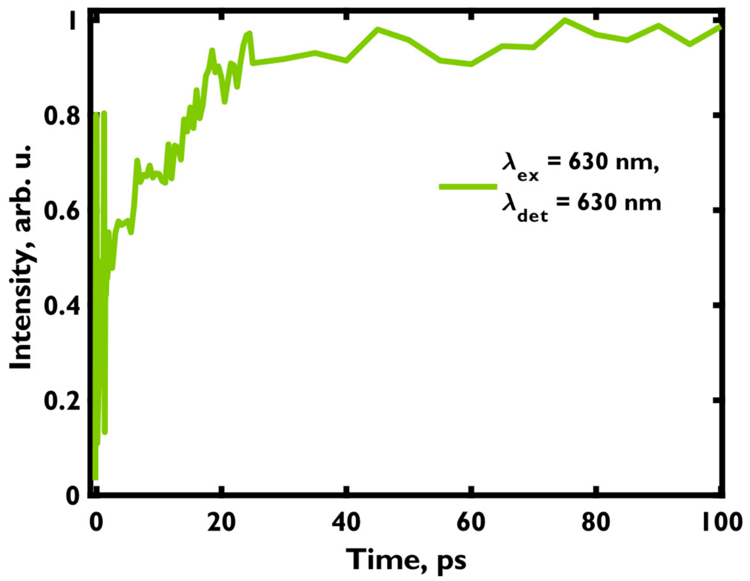

Supplementary Figure 12: Diagonal signal in the cross-peak polarized spectra.

Spectral Congestion in the Phycobilisome

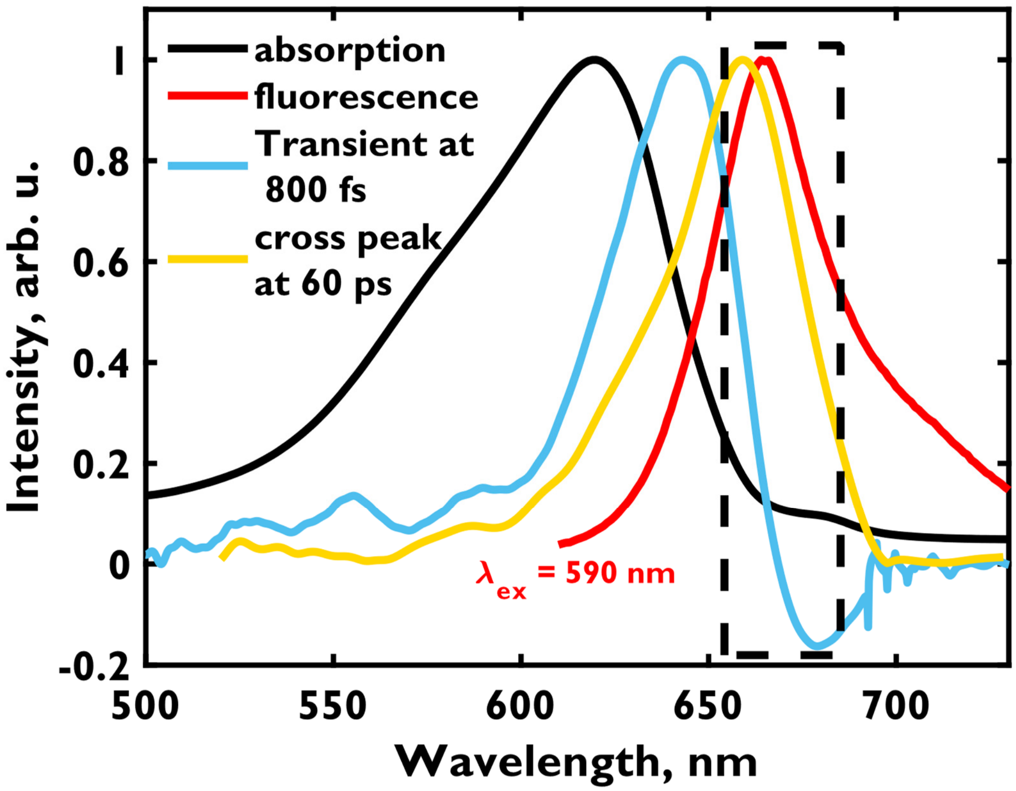

Supplementary Figure 13: A narrow spectral region between 655 and 675 nm contains peaks from on-diagonal core stimulated emission, off-diagonal excited state absorption and the cross-peak of energy transfer. All these features follow distinct (possibly multiexponential) dynamics. At late times, the comparatively large dynamic Stokes shift of the phycocyanin rods (light blue spectrum) also enters this region.

Comparison of Cross-peak Specific Dynamics with Parallel-Polarized Dynamics

Our obtained dynamics in the all-parallel data acquisition sequence match earlier reports and compartmental models yielding a sub-5 ps component, a ~50 ps component and a ~1 ns component. Similar dynamics have been observed in many studies (Refs. 15, 18, 19, 20, 21, 22, 23, 25, 26: main text) by fitting transient absorption and time-resolved emission decay traces. Our cross-peak specific spectra show distinctly different dynamics from these reports and our own all-parallel 2DES because the pulse sequence selects the cross-peak signal, or the stimulated emission from energy transfer while suppressing the highly wavelength-dependent dynamics of the other three signatures. In all-parallel 2DES and transient absorption, the time-dependent change of signal intensity is a convolution of the different dynamics of all these processes making the selective isolation of energy transfer unreliable. The large background of the diagonal signal overwhelms the dynamic response in transient absorption and all-parallel 2DES.
